# Rivaroxaban Ameliorates Sunitinib-Induced Injury of Cardiomyocytes via Repressing MAPK Signaling Pathway

**DOI:** 10.1155/cdr/2208110

**Published:** 2025-07-25

**Authors:** Ying Qian, Fang Yi

**Affiliations:** ^1^Department of Oncology, Yichang Central People's Hospital, The First College of Clinical Medical Science, China Three Gorges University, Yichang, Hubei Province, China; ^2^Tumor Prevention and Treatment Center of Three Gorges University and Cancer Research Institute of Three Gorges University, Yichang, Hubei Province, China

**Keywords:** cardiomyocytes, MAPK pathway, rivaroxaban, sunitinib

## Abstract

**Background:** Sunitinib (SU) is used to treat kidney cancer. However, it can also cause cardiotoxicity. This study is performed to investigate whether rivaroxaban (RIV) attenuates SU-induced cardiotoxicity (SIC).

**Methods and Materials:** AC16 cells and primary cardiomyocytes of neonatal mouse were treated with different concentrations (2–10 *μ*M) of SU for 24 h or with 6 *μ*M SU and 10 *μ*g/mL RIV for 24 h. The viability of cardiomyocytes was evaluated using the cell counting kit-8 (CCK-8) assay, and the apoptosis rate was evaluated using flow cytometry. The activity of caspase-3 was determined. The levels of malondialdehyde (MDA), glutathione (GSH), and superoxide dismutase (SOD) were also measured. The potential targets and downstream pathways of RIV in SIC treatment were investigated using network pharmacology, molecular docking, and molecular dynamics simulation. qPCR and western blotting were used to detect the regulatory effects of SU and RIV on mRNA and protein expression of MAPK pathway-related genes, respectively.

**Results:** RIV treatment alleviated SU-induced cardiomyocyte injury by promoting viability and inhibiting apoptosis, oxidative stress, and the inflammatory response in AC16 cells and primary cardiomyocytes. Caspase 3 (CASP3), signal transducer and activator of transcription 3 (STAT3), SRC proto-oncogene, nonreceptor tyrosine kinase (SRC), ATP-binding cassette subfamily G member 2 (ABCG2), and ATP-binding cassette subfamily B member 1 (ABCB1) were candidate targets of RIV in SIC. The binding affinities between RIV and CASP3, STAT3, SRC, ABCG2, and ABCB1 were all less than −7.5 kcal/mol, indicating that RIV could bind stably to these targets. Bioinformatics analyses suggested that the mitogen-activated protein kinase (MAPK) pathway was involved in the mechanism by which RIV alleviated SIC. RIV treatment decreased the mRNA expression of CASP3 and increased the mRNA expression of STAT3, SRC, ABCG2, and ABCB1 in AC16 cells and primary cardiomyocytes. RIV also inhibited the SU-induced activation of the MAPK pathway.

**Conclusion:** RIV exerts a protective effect against SU-induced cardiomyocyte injury by inhibiting the MAPK signaling pathway. RIV therapy may be a promising strategy to inhibit SU's cardiotoxicity in cancer patients.

## 1. Introduction

Sunitinib (SU) is a novel multitarget tyrosine kinase inhibitor (TKI) [1]. However, SU may lead to unexpected adverse effects; the more serious of which is cardiotoxicity [2]. This effect has hindered the widespread application of SU in the clinic. It is of great significance to develop effective therapeutic agents to prevent/alleviate SU-induced cardiotoxicity (SIC).

Rivaroxaban (RIV) is a selective FXa inhibitor derived from zolidinone that acts on the active site of Factor Xa (FXa) and is a novel anticoagulant drug with high safety [3]. RIV is often used for preventing thrombosis and stroke, treating deep venous thrombosis and pulmonary embolism, improving prognosis, and reducing mortality of patients with coronary heart disease [4–7]. Interestingly, some previous studies suggest RIV has organ protection effects. Specifically, RIV treatment can reduce the myocardial infarction size in rats with ischemia-reperfusion injury and also inhibit cardiomyocyte apoptosis induced by hypoxia–reperfusion (H/R) [8]. It is noteworthy that RIV exerts an inhibitory effect on oxidative stress-induced apoptosis and inflammation, thereby fulfilling a protective function in the context of SU-induced kidney injury [9]. However, the potential effects of RIV in SIC have not been fully clarified.

Network pharmacology is rapidly developing based on bioinformatics, systems biology, and pharmacology [10]. Conventional pharmaceutical design adheres to the principle of identifying the most selective ligand to interact with a singular therapeutic target [11]. Unfortunately, numerous drug development cases that have failed in Phase II and Phase III clinical trials suggest that the synergistic effect of multiple targets in diseases may be the key to finding effective ligands [10]. Consequently, the objective of this research is to elucidate the protective effect of RIV on SIC through the application of network pharmacology, in conjunction with molecular docking and experimental validation, and attempts to elucidate the possible mechanisms behind it. Using in vitro AC16 cells and primary cardiomyocytes from neonatal mice, we found that RIV alleviated SU-induced cardiomyocyte injury. This work may lay the foundation for the clinical application of RIV in alleviating SIC, which in turn benefits more patients with cancer.

## 2. Materials and Methods

### 2.1. Cell Culture and Processing

All experimental protocols were approved by the Experimental Animal Ethics Committee of Yichang Central People's Hospital. Human cardiomyocyte AC16 cells (ATCC, Rockville, Maryland, United States) were cultivated in Dulbecco's Modified Eagle Medium (DMEM) (Gibco, Walthan, Massachusetts, United States) supplemented with 10% fetal bovine serum (FBS) (Hyclone, Logan, Utah, United States), in a humidified incubator at 37°C in 5% CO_2_. Primary cardiomyocytes were obtained from the hearts of 1- to 2-day-old neonatal mice [12]. Briefly, the hearts were isolated sterile conditions, anatomically rinsed and treated in HEPES buffered saline solution (HBSS) (Gibco) chopped in medium. The tissues were digested with 0.1% Collagenase I in HBSS at 37°C for 1 h. The cell suspension was centrifuged (1000 rpm, 5 min), then resuspended in DMEM containing 10% FBS, 100 U/mL penicillin, and 100 *μ*g/mL streptomycin, and then routinely cultured. Oxygen–glucose deprivation/reoxygenation (OGD/R) model was used to induce the injury of cardiomyocytes, mimicking the patients with poor cardiac function. Briefly, the cells were cultured in DMEM without glucose, and the plates were placed in an incubator which contained a mixture of nitrogen (95%) and oxygen (5%) and cultured for 12 h. Then, the medium was discarded, DMEM containing glucose was added, and cells were cultivated in normoxia for 12 h. SU and RIV were acquired from Sigma-Aldrich (St. Louis, Missouri, United States). To assess the cytotoxicity of SU or RIV to the cells, the cells were treated with SU (2, 4, 6, 8, and 10 *μ*M) or RIV (0.625, 1.25, 2.5, 5, and 10 *μ*g/mL) for 24 h. To investigate the protective effect of RIV on SU-induced cardiomyocyte injury, 6 *μ*M SU treatment and 10 *μ*g/mL RIV treatment for 24 h were performed.

### 2.2. Cell Counting Kit-8 (CCK-8) Assay

The cardiomyocytes in logarithmic stages were inoculated in 96-well plates (1 × 10^3^ cells/mL, 100 *μ*L cell suspension/well). The cells were cultivated for 24 h. Subsequently, 10 *μ*L of CCK-8 solution (Dojindo, Shanghai, China) was added to each well, and the cells were incubated at 37°C for 2 h. Then, the absorbance values of the wells at 450 nm were measured with an enzyme-labeled instrument.

### 2.3. Evaluation of Caspase-3 Activity

A caspase-3 activity assay kit (Beyotime, Shanghai, China) was used to assess the activity of caspase-3 of the cardiomyocytes in different groups. The cells were lysed, and the total protein was extracted. The total protein of the cells was determined using a BCA protein assay kit (Beyotime) and then incubated at 37°C overnight with an equal amount of Ac-DEVD-pNA to determine caspase-3. Next, enzyme-linked immunosorbent assay (ELISA) was used to detect the absorbance at 405 nm to assess pNA release. Caspase-3 activity was calculated as mean absorbance/mean control absorbance × 100%.

### 2.4. Flow Cytometry

An Annexin V-FITC/PI apoptosis detection kit (Beyotime) was applied. Briefly, the cells were trypsinized, centrifuged (1000 rpm, 5 min) and washed three times with phosphate buffer saline (PBS). The cells were then added in 100 *μ*L 1× binding buffer and stained with 5 *μ*L Annexin V-FITC and 5 *μ*L PI in the dark for 10 min. After the cells were washed with PBS again, 400 *μ*L 1× binding buffer was added to each sample and fully mixed. Within 1 h, a flow cytometer (BD Biosciences, San Jose, California, United States) was applied to sort the cells. FlowJo software was used to analyze the results.

### 2.5. Examination of Oxidative Stress

The cardiomyocytes were collected and centrifuged (10 min, 3000 × g, 4°C). Malondialdehyde (MDA) assay kit (Jiancheng, Inc., Nanjing, China), glutathione (GSH) assay kit (Beyotime), and superoxide dismutase (SOD) assay kit (Jiancheng, Inc.) were used to detect MDA levels, GSH, and SOD activity in the supernatant.

### 2.6. Reverse Transcriptional Quantitative Polymerase Chain Reaction (qPCR)

Total RNA of AC16 cells in different groups was extracted by TRIzol reagent (Invitrogen). Total RNA was reverse-transcribed into cDNA with a PrimeScript RT Reagent Kit (Takara, Dalian, China). Subsequently, SYBR Premix Ex Taq II (Takara) was used for DNA amplification in 7500 real-time PCR (Applied Biosystems, Foster City, California, United States). The thermal cycle conditions were as follows: 95°C for 5 min, 95°C for 30 s, 60°C for 45 s (40 cycles), and 72°C for 30 min. The sequences of the primers are shown in Table 1.

### 2.7. Western Blot

The cardiomyocytes were incubated with a cold RIPA lysis buffer (EMD Millipore Billerica, Massachusetts, United States) to extract the total protein. After mixing with loading buffer and protease inhibitor and denaturation, the protein samples were separated after SDS-PAGE and transferred onto a PVDF membrane. The membrane was blocked with 5% skim milk and then incubated with primary antibodies. After washing, goat anti-rabbit IgG H&L (Alexa Fluor 488) (ab150077, 1:1000) coupled with horseradish peroxidase was applied to incubate the membrane. Finally, an enhanced chemiluminescence detection reagent (Beyotime) was used to detect protein bands on the film. ImageLab software Version 4.1 (Bio-Rad Laboratories, Hercules, California, United States) was used for signal capture and density value calculation. The primary antibodies are as follows: anti-B-cell lymphoma 2 (Bcl-2) antibody (ab182858, 1:1000), anti-Bcl-2 associated X protein (Bax) antibody (ab182733, 1:1000), anti-extracellular signal-regulated kinase 1/2 (ERK1/2) antibody (ab184699, 1:1000), anti-phospho (p)-ERK1/2 (T202/Y204) (ab278538, 1:1000), anti-c-Jun N-terminal kinase (JNK) (ab179461, 1:1000,), anti-p-JNK (T183/Y185) (ab307802, 1:1000), anti-p38 (ab170099, 1:1000), anti-p-p38 (T180) (ab178867, 1:1000), and anti-GAPDH (ab9485, 1:1000). The antibodies were obtained from Abcam (Cambridge, United Kingdom). The original western blots were shown in the Supporting Information section.

### 2.8. Collection of RIV Targets and SIC-Related Targets

From the PubChem, the RIV canonical SMILES file was obtained, and it was imported into the SwissTaraetPrediction database, to predict the potential targets. In addition, Search Tool for Interactions of Chemicals (STITCH) database, Comparative Toxicogen Omics (CTD) database, Pharmacogenetics and Pharmacogenomics Knowledge Base database, and DrugBank database were searched to obtain additional RIV targets. SIC related targets from the human genome database (GeneCards). Specifically, in GeneCards, using precise search phrases to get a target, such as “Sunitinib-induced cardiotoxicity,” “Sunitinib-induced myocardial infarction,” “Sunitinib-induced heart failure,” “Sunitinib-induced cardiomyopathy,” and “Sunitinib-induced injury of cardiomyopathy“. After the duplicate targets were removed, the remaining targets were standardized using the Uniprot database. Then, the targets in the intersection were collected (RIV's targets in SIC treatment). Subsequently, Gene Ontology (GO) and Kyoto Encyclopedia of Genes and Genomes (KEGG) pathway enrichment analyses were performed using the R package “clusterProfiler”, with all protein-coding genes annotated in the human genome serving as the background gene set. The significance thresholds were set as raw *p* value < 0.01 and false discovery rate (FDR)–adjusted *p* value < 0.01, respectively. The Top 10 GO and KEGG results were visualized using the Weishengxin Online Platform.

### 2.9. Construction of Protein–Protein Interaction (PPI) Networks

Relevant targets of RIV in SIC treatment were imported into STRING database, the PPI network was obtained, and the TSV file was downloaded and imported into Cytoscape 3.9.0 software for cluster analysis via the MCODE plug-in. In addition, the Centiscape 2.2 plug-in was utilized to obtain the centrality index of PPI network nodes. The hub targets of RIV for SIC were screened according to betweenness centrality (BC) ≥ 6.80, closeness centrality (CC) ≥ 0.05, degree centrality (DC) ≥ 7.73.

### 2.10. Molecular Docking

The three-dimensional structure of RIV was retrieved from the PubChem database and saved in the SDF format. Subsequently, this structure was transformed into the mol2 format using OpenBabel software, Version 3.1.1. Three-dimensional structures of the hub targets were obtained from the Protein Data Bank (PDB) database. All protein receptor and molecular ligand files were converted into the pdbqt format using the AutoDockTools software, Version 1.5.7. Subsequent to this, molecular docking was conducted utilizing AutoDock Vina, Version 1.1.2, as indicated in Table 2. Binding energies below zero signify that the proteins and the drug can spontaneously engage in binding and interaction. Visualization was accomplished using the PyMOL software.

### 2.11. Molecular Dynamics Simulation (MDS)

Gromacs2022.3 software was used for MDS [13], and the procedures were performed according to a recently reported study [14]. After molecules were preprocessed and the simulation conditions were set, free MDS was performed, which lasted for 100 ns. The root-mean-square variance (RMSD) values were calculated. Additionally, the binding free energies between the receptors and ligands were evaluated with molecular mechanics/Poisson-Boltzmann/generalized born surface area (MMGBSA).

### 2.12. Statistical Analysis

The data of three or more independent assays were analyzed by Graphad Prism 9.0 software. The data that adhered to the normal distribution were denoted using the format “mean ± standard deviation”. In the figures, the dots indicate the values of replicates during the assays. Student's *t*-test or one-way analysis of variance and Tukey post hoc test were used for comparison between groups. *p* < 0.05 was considered to be significant.

## 3. Results

### 3.1. SU Induced Viability Suppression, Apoptosis, Oxidative Stress, and Inflammation of Cardiomyocytes

**Figure 1:**
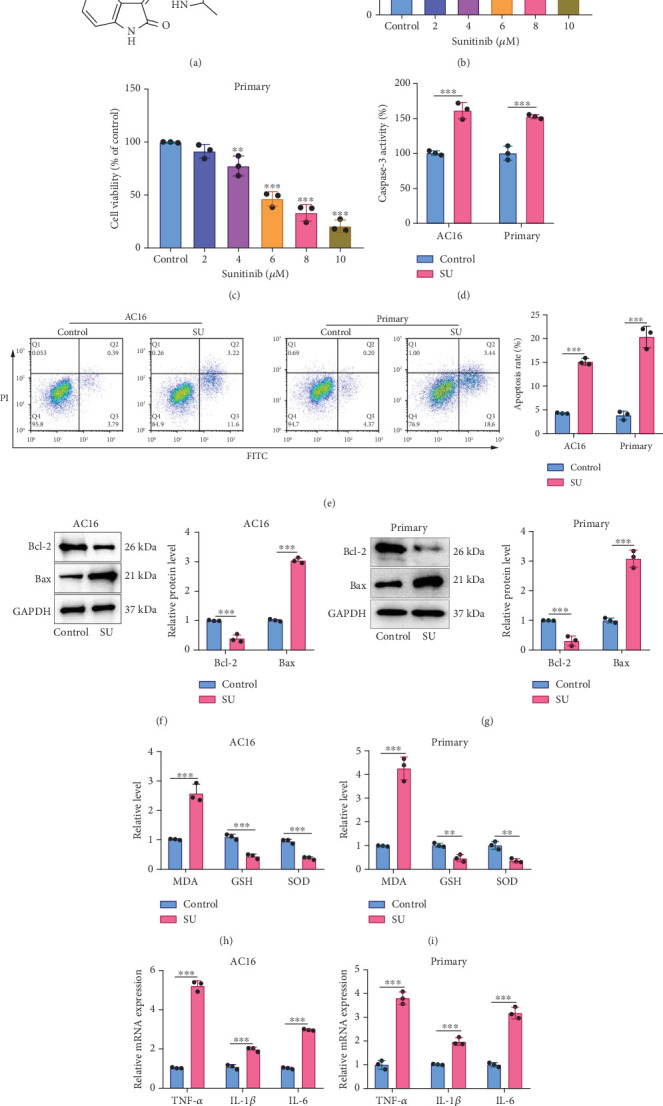
SU induces injury of cardiomyocytes. (a) Chemical structure of SU. (b, c) AC16 cells and primary cardiomyocytes were treated with different concentrations (2, 4, 6, 8, and 10 *μ*M) of SU for 24 h, and cell viability was detected by the CCK-8 method. (d) After treating AC16 cells and primary cardiomyocytes without or with 6 *μ*M SU for 24 h, the injury of AC16 cells and primary cardiomyocytes was detected by a caspase-3 assay kit. (e) Flow cytometry was used to evaluate the apoptosis rate of AC16 cells and primary cardiomyocytes. (f, g). Protein expression levels of Bcl-2 and Bax in AC16 cells and primary cardiomyocytes were detected by western blot. (h, i) The levels of MDA, GSH, and SOD in AC16 cells and primary cardiomyocytes were detected to evaluate oxidative stress. (j, k) qPCR was used to detect the mRNA expression levels of inflammatory factors including TNF-*α*, IL-1*β*, and IL-6 in AC16 cells and primary cardiomyocytes. All of the experiments were performed in triplicate and repeated for at least three times. The dots in the graphs are representative of three independent biological experiments. ∗∗ and ∗∗∗ represent *p* < 0.01 and *p* < 0.001, respectively.

SU's chemical structure is shown in Figure 1a. To determine the cytotoxicity of SU to AC16 cells and primary cardiomyocytes, after OGD/R, the cells were treated with different concentrations of SU for 24 h. SU could reduce the viability of cardiomyocytes in a dose-dependent manner (Figure 1b,c). The IC_50_ of AC16 cells and primary cardiomyocytes treated with SU for 24 h were 5.098 and 5.973 *μ*M, respectively. Subsequently, the cells were treated with 6 *μ*M of SU for 24 h. It was observed that SU treatment significantly enhanced the caspase-3 protease activity (Figure 1d). Flow cytometry showed that SU treatment increased the rate of apoptosis of AC16 cells and primary cardiomyocytes (Figure 1e). Western blot showed that after SU treatment, the expression level of Bcl-2 protein in cardiomyocytes was decreased, while the expression level of Bax protein was increased (Figure 1f,g). In addition, MDA levels in the cardiomyocytes in the SU treatment group were increased, while GSH and SOD levels were decreased (Figure 1h,i), suggesting SU induced oxidative stress response of cardiomyocytes. qPCR showed that SU treatment markedly increased the mRNA expression levels of TNF-*α*, interleukin-1*β* (IL-1*β*), and interleukin-6 (IL-6) in the cells (Figure 1j,k), suggesting SU induces inflammatory response of cardiomyocytes. These results imply that SU treatment may induce myocardial injury, especially for the patients with heart diseases.

### 3.2. RIV Reversed SU-Induced Injury of Cardiomyocytes

**Figure 2:**
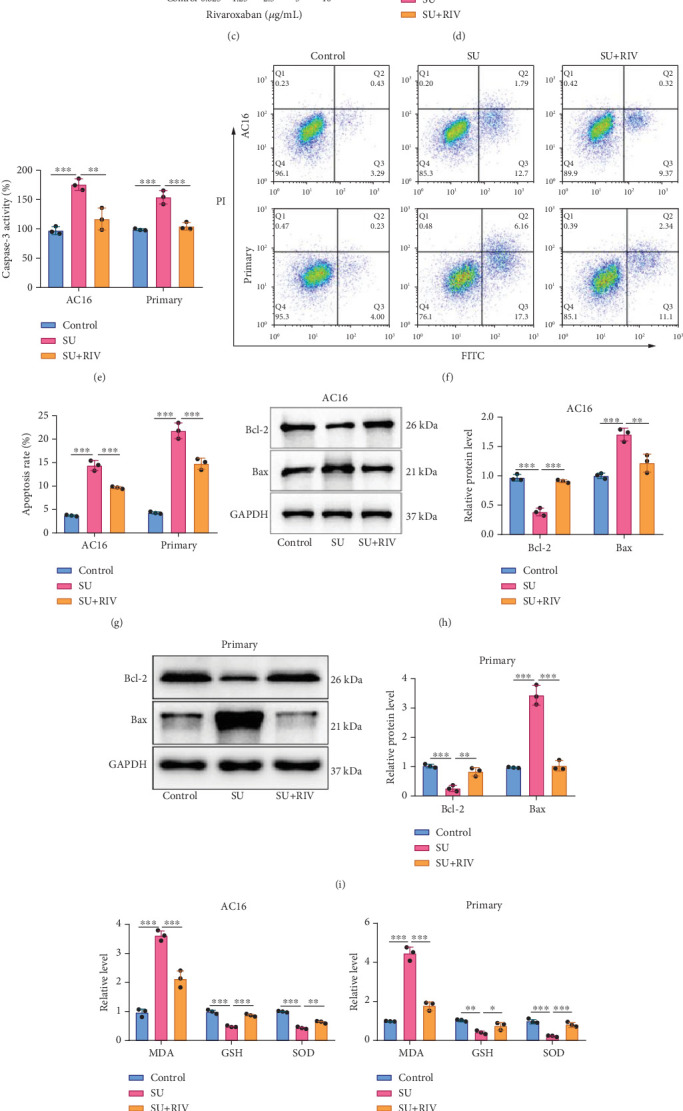
RIV treatment reverses SU-induced injury of cardiomyocytes. (a). Chemical structure of RIV. (b, c) AC16 cells and primary cardiomyocytes were treated with RIV at different concentrations (0.625, 1.25, 2.5, 5, and 10 *μ*g/mL) for 24 h, and cell viability was detected by the CCK-8 method. (d). After treating AC16 cells and primary cardiomyocytes with 6 *μ*M SU or/and 10 *μ*g/mL RIV for 24 h, the cell viability was assessed by the CCK-8 method. (e). A caspase-3 assay kit was used to detect the activity of caspase-3 in AC16 cells and primary cardiomyocytes. (f, g) The apoptosis of AC16 cells and primary cardiomyocytes was detected by flow cytometry. (h, i) Protein expression levels of Bcl-2 and Bax in AC16 cells and primary cardiomyocytes were detected by Western blot. (j, k) The levels of MDA, GSH, and SOD in AC16 cells and primary cardiomyocytes were detected by the corresponding detection kit. (l, m) qPCR was used to detect the mRNA expression levels of inflammatory factors, including TNF-*α*, IL-1*β*, and IL-6 in AC16 cells and primary cardiomyocytes. All of the experiments were performed in triplicate and repeated at least three times. The dots in the graphs are representative of three independent biological experiments. ∗, ∗∗, and ∗∗∗ represent *p* < 0.05, *p* < 0.01, and *p* < 0.001, respectively.

The chemical structure formula of RIV is shown in Figure 2a. To prove the low toxicity of RIV on cardiomyocytes, the effect of RIV treatment on the viability of AC16 cells and primary cardiomyocytes was first evaluated. CCK-8 assay showed that when the cardiomyocytes were treated with RIV concentration ≤ 10* μ*g/mL, the cell viability was not markedly changed (Figure 2b,c). Ten micrograms per milliliter RIV was selected for follow-up experiments. It was observed that RIV treatment markedly reversed SU-induced inhibition of the viability of the cardiomyocytes (Figure 2d). In addition, caspase-3 activity in the cardiomyocytes in the SU + RIV group was markedly reduced compared to the SU group (Figure 2e). RIV treatment inhibited SU-induced apoptosis of AC16 cells and primary cardiomyocytes (Figure 2f,g). Western blot results showed that RIV treatment markedly reversed the downregulation of Bcl-2 protein expression level and upregulation of Bax protein expression level induced by SU treatment (Figure 2h,i). In addition, MDA levels and mRNA expression levels of inflammatory factors (TNF-*α*, IL-6, and IL-1*β*) were markedly decreased in AC16 cells and primary cardiomyocytes treated with SU and RIV compared with the SU treatment group, while GSH and SOD levels were remarkably increased (Figure 2j,m). These results suggest that RIV protects cardiomyocytes from the injury induced by SU.

### 3.3. Functional Enrichment Analysis of RIV's Targets in SIC Treatment

**Figure 3:**
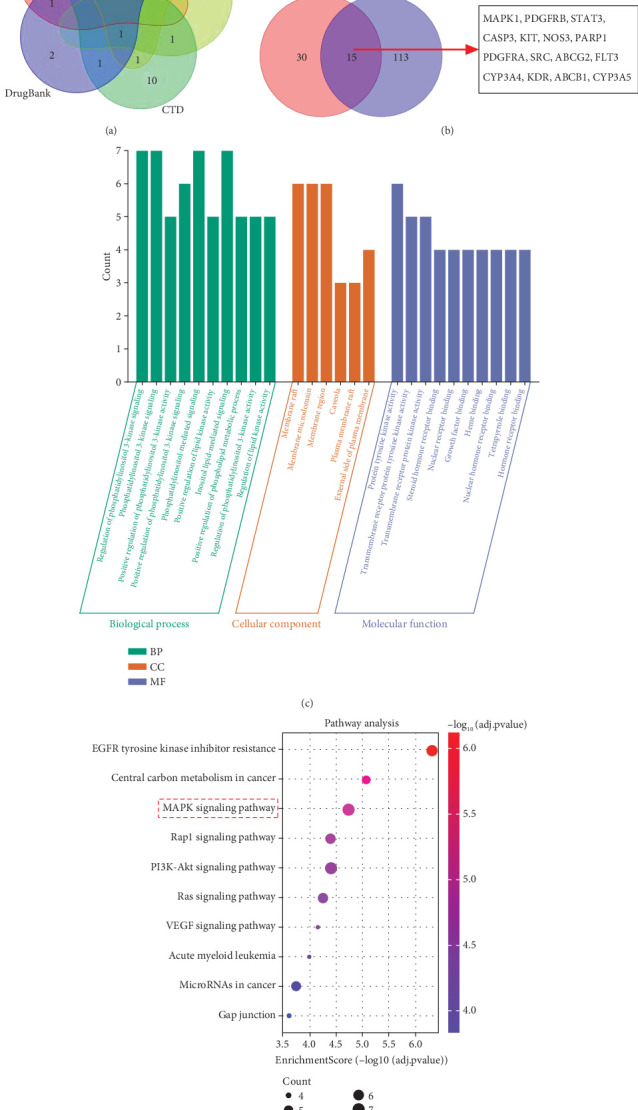
GO and KEGG enrichment analysis of candidate targets of RIV in SIC treatment. (a). Collection of RIV's targets. (b). The venn diagram of RIV's targets and SIC-related genes. (c). GO enrichment analysis of RIV's targets in SIC treatment. Biological process (BP) is marked by dark cyan, cellular component (CC) is marked by sienna, and molecular function (MF) is marked by steel blue. (d). Bubble map of KEGG pathway enrichment analysis of RIV's targets in SIC treatment. The bubble size represents count, and the bubble color represents the *p* value. MAPK pathway is one of the significant pathways probably modulated by these targets.

A total of 102, 7, 7, 15, and 6 targets were obtained from SwissTargetPrediction, PharmGKB, STITCH, CTD, and DrugBank databases, respectively, and a total of 128 RIV targets were obtained (Figure 3a). Additionally, the GeneCards database provided 45 SIC-related target genes, and a total of 15 candidate targets in the intersection were regarded as the target genes of RIV in SIC treatment (Figure 3b). In GO analysis, the Top 10 biological processes (BPs), cellular components (CC), and molecular functions (MF) were selected for visualization, respectively (Figure 3c). Also, the Top 10 pathways in KEGG analysis were visualized (Figure 3d). The detailed results of these analyses are shown in the supporting tables (false FDR-adjusted *p* value < 0.01). These functional annotation analyses suggested that these targets were associated with the PI3K/AKT pathway, MAPK pathway, etc.

### 3.4. Identification of Hub Targets

**Figure 4:**
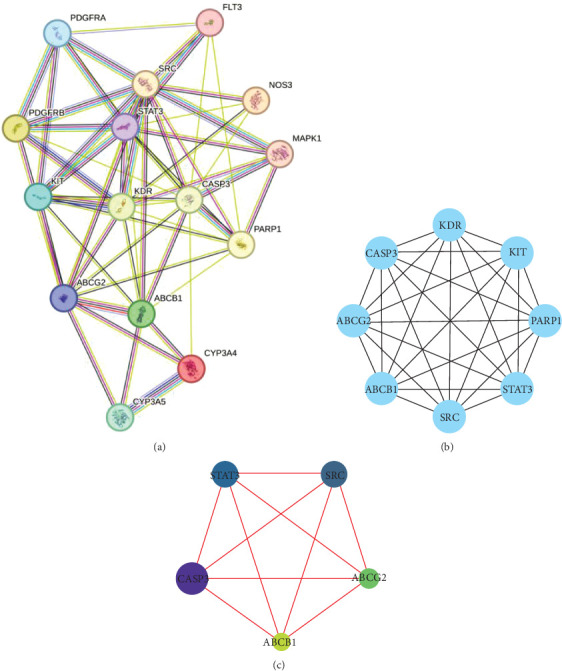
PPI network construction and analysis of RIV treatment SIC candidate targets. (a) A PPI network of RIV's targets in SIC treatment was constructed based on String database. Nodes represent proteins, and edges represent protein–protein interactions. The olivine edges indicate the interactions from text mining. The black edges indicate coexpression. The purple edges indicate the interactions which were determined experimentally. The blue edges indicate the interactions with gene co-occurrence. The azury edges indicate the interactions from curated databases. The lilac edges indicate protein homology. More details can be found in STRING database (https://cn.string-db.org/). (b) Cluster analysis of PPI network with MCODE plug-in. (c) Centiscape 2.2 plug-in was used to obtain the hub nodes in PPI network. The size of the node is proportional to the size of the degree value.

In order to explore hub targets of RIV in SIC treatment, a comprehensive network analysis was constructed based on the 15 candidate targets, and a PPI network containing 15 nodes and 58 edges was retrieved (Figure 4a). Subsequently, the MCODE plug-in was used to perform cluster analysis on the PPI network and generate a highly connected gene cluster (Figure 4b). In addition, the node-based BC, CC, and DC values were applied to obtain a pivotal network which consisted of five hub genes (Figure 4c and Table 2). Accordingly, CASP3, STAT3, SRC, ABCG2, and ABCB1 were considered to be hub targets for RIV in SIC treatment.

### 3.5. Molecular Docking and MDS

**Figure 5:**
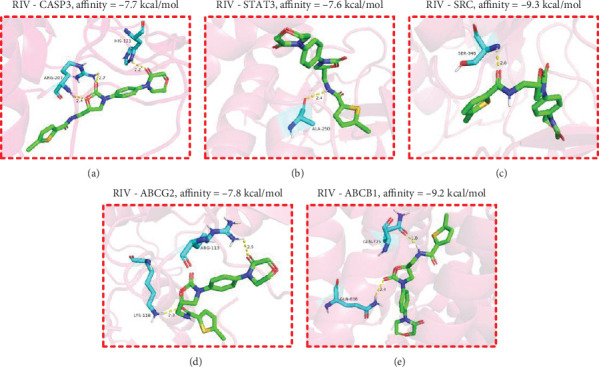
Molecular docking between RIV and hub targets. (a–e) Molecular docking diagram of RIV with (a) CASP3, (b) STAT3, (c) SRC, (d) ABCG2, and (e) ABCB1 proteins. Light blue indicates amino acid residues surrounding the binding bag, green indicates RIV, pink indicates macromolecules, and yellow dashed lines indicate hydrogen bonding.

**Figure 6:**
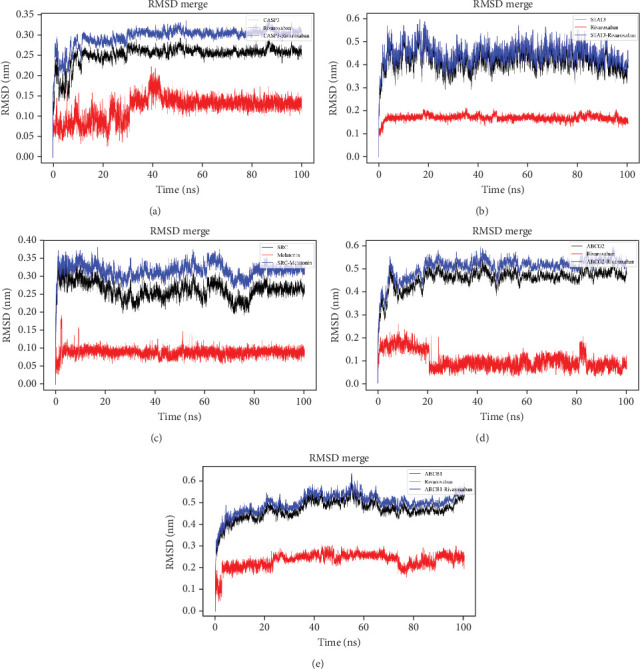
MDS suggests that RIV binds stably with the hub targets. Evolution of RMSD values during 100 ns MDS of (a) RIV/CASP3 complex, (b) RIV/STAT3 complex, (c) RIV/SRC complex, (d) RIV/ABCG2 complex, and (e) RIV/ABCB1 complex.

Molecular docking was conducted to investigate the binding affinity between the hub targets and RIV (Table 3). As shown, RIV could probably form multiple hydrogen bonds with amino acid residues of CASP3 protein, STAT3 protein, SRC protein, ABCG2 protein, and ABCB1 protein (Figures 5a, 5b, 5c, 5d, and 5e). These results suggest that RIV binds stably to the hub targets, suggesting that it exerts its cardioprotective effects via regulating CASP3, STAT3, SRC, ABCG2, and ABCB1. Then, MDS was performed. All of the RMSD values of the complexes, which were formed by RIV and the hub targets, increased rapidly and reached states of equilibrium in 20 ns, and after equilibrium, the change of RMSD values was less than 0.2 nm, also implying that the binding relationships were highly stable (Figures 6a, 6b, 6c, 6d, and 6e). The MMGBSA binding free energy analysis of RIV and these hub targets indicated that the van der Waals interactions and gas-phase energy were the main driving forces for binding, suggesting that hydrophobic interactions played a main role in the stability of the complexes. Electrostatic interactions also provided moderate support for the stability of the complex stabilities. Although solvation energy counteracted the binding, the overall binding free energies were negative, indicating that the binding of RIV to these candidate targets was thermodynamically favorable, primarily driven by hydrophobic effects and electrostatic interactions (Table 4).

### 3.6. RIV Inhibited MAPK Signaling Activation in Cardiomyocytes Induced by SU

**Figure 7:**
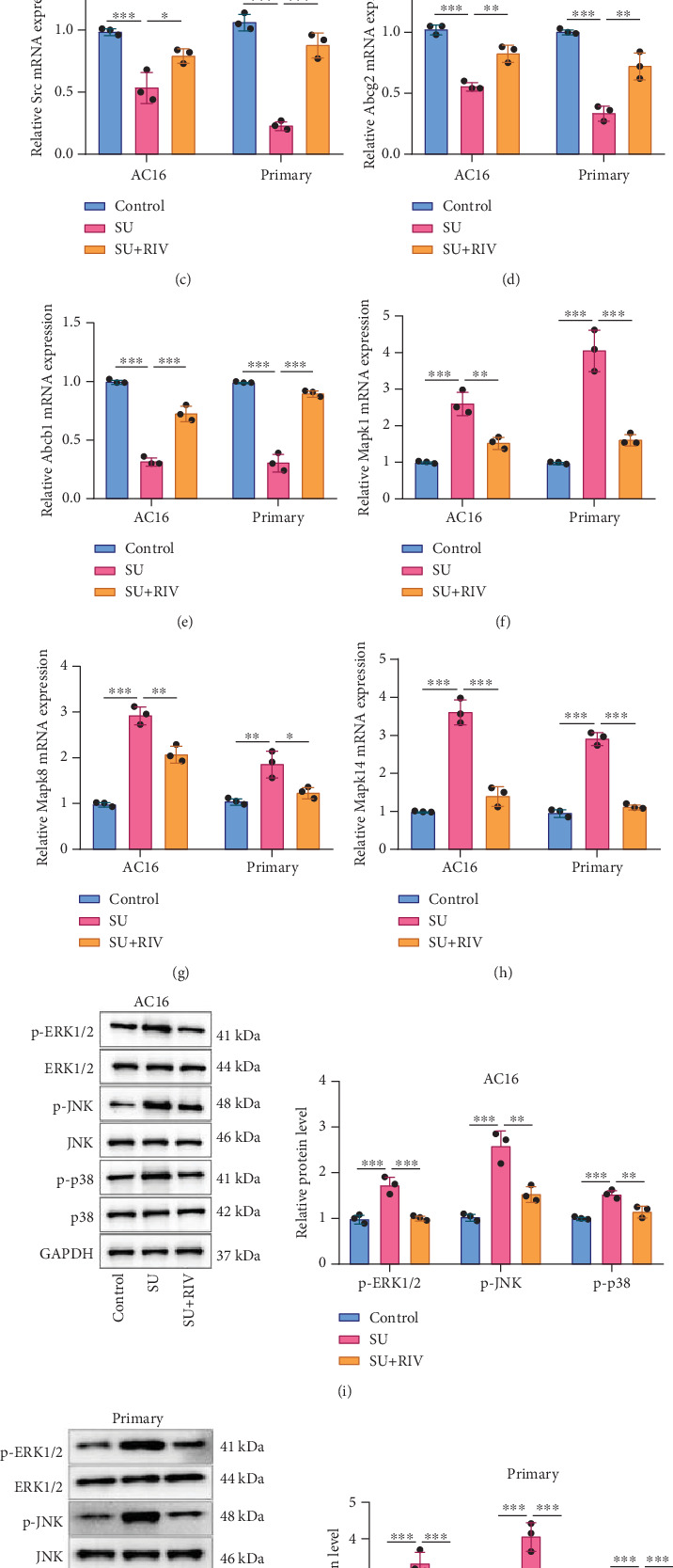
Effects of RIV on the expression levels of hub genes and MAPK pathway-related genes in SU-induced cardiomyocytes. (a–h) After treating AC16 cells and primary cardiomyocytes with 6 *μ*M SU and/or 10 *μ*g/mL RIV for 24 h, the mRNA expression levels of (a) CASP3, (b) STAT3, (c) SRC, (d) ABCG2, (e) ABCB1, (f) MAPK1, (g) MAPK8, and (h) MAPK14 were detected by qPCR. (i, j) Protein expression levels of p-ERK1/2, p-JNK, and p-p38 were detected by Western blot. All of the experiments were performed in triplicate and repeated for at least three times. The dots in the graphs are representative of three independent biological experiments. ∗, ∗∗, and ∗∗∗ represent *p* < 0.05, *p* < 0.01, and *p* < 0.001, respectively.

To verify whether the protective effect of RIV on SU-induced cardiomyocyte was mediated by the hub genes mentioned above, the mRNA expression levels of hub targets were measured. SU treatment markedly upregulated CASP3 mRNA expression levels and decreased STAT3, SRC, ABCG2, and ABCB1 mRNA expression levels in AC16 cells and primary cardiomyocytes, while RIV treatment remarkably reversed these effects (Figures 7a, 7b, 7c, 7d, and 7e). We also analyzed the effect of RIV on the MAPK signaling pathway in SU-induced AC16 cells and primary cardiomyocytes. qPCR demonstrated that SU treatment promoted the mRNA expression levels of MAPK1, MAPK8, and MAPK14 in AC16 cells and primary cardiomyocytes, while RIV treatment reversed this effect (Figures 7f, 7g, and 7h). Also, SU treatment increased protein levels of p-ERK1/2, p-JNK, and p-p38 in AC16 cells and primary cardiomyocytes, while RIV treatment attenuated these effects (Figure 7i,j).

## 4. Discussion

SU has shown definite antitumor effects in patients with advanced renal cell carcinoma and gastrointestinal stromal tumor who are resistant to imatinib [15, 16]. However, SU sometimes leads to severe cardiac side effects in clinic, including hypertension, systolic dysfunction, and congestive heart failure, probably caused by the injury of cardiomyocytes [1, 17–19]. Consequently, it is of paramount importance to devise innovative strategies to avert SIC without undermining its efficacy in combating cancer. In this study, we proved that SU can induce myocardial cell damage by reducing the survival rate of cardiomyocytes and promoting cell apoptosis, which is consistent with previous studies [20, 21]. Bcl-2 is an important anti-apoptotic gene, while Bax promotes apoptosis [22]. Our study found that SU induced a decrease in Bcl-2 expression and an increase in Bax expression, suggesting that SU might be involved in cardiomyocyte apoptosis by inhibiting Bcl-2 and promoting Bax expression. In addition, SU induced oxidative stress and inflammation in cardiomyocytes.

As an oral anticoagulant, RIV has the advantages of rapid onset, high efficacy, and high safety [23, 24]. Importantly, emerging studies suggest that RIV has cardioprotective effects. RIV enhances cellular viability under conditions of hypoxia by suppressing the protease-activated receptor 2 signaling pathway; it diminishes the expression of molecules associated with inflammation and fibrosis in cardiac fibroblasts, thereby reducing the incidence of aortic atherosclerosis and coronary artery occlusion, and it significantly mitigates cardiac fibrosis [25]. RIV can also reduce the expression levels of PAR-1, PAR-2, TNF-*α*, and TGF-*β*, thereby preventing cardiac insufficiency in a mouse model of myocardial infarction and attenuating cardiac remodeling [26]. In this study, we report that RIV can reverse SU-induced viability inhibition and apoptosis of cardiomyocytes. RIV can also reduce the level of MDA and the mRNA expression of inflammatory factors (TNF-*α*, IL-1*β*, and IL-6) and increase the level of GSH and SOD, thereby alleviating the SU-induced oxidative stress and inflammatory response. This suggests that RIV may be a potential protective agent for cardiotoxicity of SU. Notably, a previous study reports that RIV ameliorates SIC by inhibiting oxidative stress-mediated inflammation in a rat model [27]. In the present work, both human cardiomyocyte cell line AC16 and primary cardiomyocytes from neonatal mouse were used, and the results are consistent with the previous study [27], which further validates the effects of RIV in protecting the heart from SIC.

Based on network pharmacological analysis, in the present work, five hub targets for RIV against SIC were identified, including CASP3, STAT3, SRC, ABCG2, and ABCB1. CASP3 is a protease that plays a key role in apoptosis, and inhibiting the activity of CASP3 can reduce the apoptosis of cardiomyocytes [28, 29]. STAT3 is a cellular signal–transducing protein that has the capacity to facilitate cardiomyocyte proliferation, angiogenesis, and extracellular matrix homeostasis, while also diminishing the cardiotoxicity of adriamycin [30, 31]. Transient activation of STAT3 has been reported to be beneficial and protective to the heart [32]. SRC is a nonreceptor tyrosine kinase that has prosurvival effects in cardiomyocytes [33]. Overexpression of c-Src T338I has been reported to salvage the toxicity of dasatinib to cardiomyocytes [34]. The protective effect of metformin against doxorubicin-induced H9c2 cardiomyocyte toxicity was lost due to SRC knockdown [35]. ABCG2 and ABCB1 are members of the ATP-binding box (ABC) membrane transporter superfamily [36, 37]. ABCG2 and ABCB1 are pharmacokinetic-related genes of SU [38, 39]. ABCG2 rs2231142 polymorphism can be used as a predictor of SU-induced thrombocytopenia and hand-foot syndrome in Asian people, while ABCB1 rs1128503 polymorphism can be used as a predictor of SU-induced hypertension [39]. In addition, ABCG2 can prevent cardiac hypertrophy and heart failure caused by pressure overload [40], and suppression of inherent ABCB1 elevates the susceptibility to doxorubicin-induced cardiotoxicity [41]. Molecular docking in the present work showed that RIV can stably bind to CASP3, STAT3, SRC, ABCG2, and ABCB1, suggesting that it may be a potential drug to target these targets. In addition, our data showed that SU treatment increased mRNA expression levels of CASP3 and decreased mRNA expression levels of STAT3, SRC, ABCG2, and ABCB1 in cardiomyocytes, while RIV treatment significantly reversed these effects. These findings suggest that RIV may play a protective role in SIC by acting on CASP3, STAT3, SRC, ABCG2, and ABCB1. Of course, the detailed regulatory functions of RIV on these target genes are necessary to be studied in the following work to further decipher the role of RIV on attenuating the injury of cardiomyocytes.

The previous study mentioned above indicates that RIV exerts its cardioprotective effects probably via TGF*β*/Smad pathway [27]. However, the underlying mechanism is not investigated in human cells, but in a rat model, and the detailed mechanism of RIV in protecting cardiomyocytes still requires exploration. The data in our work suggest that, in human cardiomyocytes, the MAPK pathway may be the key pathway for RIV in SIC treatment: It was revealed that MAPK1 was among the 15 candidate targets of RIV in SIC treatment, and KEGG enrichment analysis also suggested that MAPK signaling was among the pathways with the highest significance. The MAPK pathway plays a key role in various BPs such as cell growth, differentiation, migration, and apoptosis [42]. The proteins in the MAPK pathway, including ERK1/2, JNK, and p38 MAPK, are activated in response to extracellular signals and transmit the signals to the nucleus, thereby affecting gene expression [43–45]. In cardiomyocytes, abnormal activation or inhibition of MAPK signaling is associated with the pathogenesis of heart disease. For example, setanaxib can inhibit the activation of the MAPK pathway by reducing the phosphorylation levels of JNK, ERK, and p38, thereby inhibiting cardiomyocyte apoptosis [46]. Resveratrol inhibits ferroptosis by regulating MAPK signaling, thus alleviating adriamycin-induced cardiotoxicity [47]. In addition, a previous study reports that SU induces hypertrophy of rat cardiomyocyte H9c2 cells in a MAPK-dependent mechanism [48]. The MAPK signaling pathway exhibits a bifurcated function, serving as either an activator or an inhibitor, contingent upon the cellular context and the nature of the stimulus [49]. Increased p38 phosphorylation leads to distinct biological effects in different conditions. A previous study has reported that p38 can phosphorylate and inhibit the activities of caspase-8 and caspase-3 and promote the survival of neutrophils [50]. However, in cardiomyocytes, the prevailing view supports that p38 phosphorylation promotes the apoptosis and injury of cardiomyocytes [49, 51–53], probably via modulating p53 and Bcl2 family proteins. Interestingly, this study found that SU can upregulate the protein levels of p-ERK1/2, p-JNK, and p-p38 in AC16 cells and primary cardiomyocytes and can also increase the mRNA expression levels of MAPK1, MAPK8, and MAPK14, thus leading to the activation of the MAPK pathway. The combination of RIV and SU inhibited the activation of the MAPK pathway. This suggests that the protective effect of RIV on SU-induced cardiomyocyte injury may be related to the regulation of the MAPK pathway. Interestingly, RIV has been reported to repress angiotensin II-induced activation of cardiac fibroblasts, partly via inhibiting MAPK signaling [54]. Considering cardiac fibroblasts are the main contributors in promoting cardiac fibrosis [54], RIV may also repress MAPK signaling in fibroblasts to mitigate SU-induced heart function deterioration, and this hypothesis remains to be verified in the future. Notably, our analysis suggested that the PI3K/AKT pathway may also be a crucial effector of RIV in protecting cardiomyocytes. The dysregulation of the PI3K/AKT pathway is associated with a series of heart disorders [55]. Even though there are no reports suggesting SU treatment regulates the PI3K/AKT pathway to induce heart injury, some studies have demonstrated that SU represses PI3K/AKT signaling [56, 57]. Additionally, the PI3K/AKT pathway and the MAPK pathway work collaboratively within cells to regulate multiple BPs [58, 59]. The regulatory effects of RIV on PI3K/AKT signaling in its cardioprotective functions deserve further investigation in the following work.

There are several limitations in the present work. This study demonstrated that RIV may exert biological effects on CASP3, STAT3, SRC, ABCG2, and ABCB1 through molecular docking and MDS. It is worth noting that molecular docking and MDS are in silico analyses. In subsequent studies, the regulatory effect of RIV on these proteins needs to be further verified by means of molecular biology. In addition, it has been reported in the past that these core proteins are not only related to damaging factors such as oxidative stress and inflammation but also interact with the MAPK pathway [60–62]. Whether RIV can affect MAPK signaling by regulating the activity of these proteins is also worth exploring in the future. This is particularly important for further elucidating the molecular mechanism by which RIV protects cardiomyocytes. Finally, animal models remain to further validate the findings in subsequent studies.

## 5. Conclusion

RIV enhances cardiomyocyte viability, inhibits apoptosis, suppresses oxidative stress, and represses inflammatory response by inhibiting the MAPK pathway, thereby alleviating SU-induced cardiomyocyte injury. RIV may be a potential cardioprotectant for SIC. In the future, clinical trials are needed to confirm the findings in this study.

## Figures and Tables

**Table 1:** Sequences of the primers used for qPCR.

**Gene**	**Human (5**⁣′**→3**⁣′**)**	**Mouse (5**⁣′**→3**⁣′**)**
TNF-*α*	F: CACAGTGAAGTGCTGGCAAC	F: ATGGCCTCCCTCTCATCAGT
	R: ACATTGGGTCCCCCAGGATA	R: TTTGCTACGACGTGGGCTAC
IL-1*β*	F: AACCTCTTCGAGGCACAAGG	F: AGGTCAGTGGGTACTGGAGAG
	R: AGATTCGTAGCTGGATGCCG	R: CCTTTGCTTCCAAGCCAGAC
IL-6	F: CCACCGGGAACGAAAGAGAA	F: GCCTTCTTGGGACTGATGCT
	R: TGTTACATGTTTGTGGAGAAGGA	R: TGTGACTCCAGCTTATCTCTTGG
CASP3	F: GCTCATACCTGTGGCTGTGT	F: GGGGAGCTTGGAACGCTAAG
	R: GCTTTGGTTCCCGCAAAACT	R: CCGTACCAGAGCGAGATGAC
STAT3	F: TCTGCCGGAGAAACAGTTGG	F: ACCAACGACCTGCAGCAATA
	R: AGGTACCGTGTGTCAAGCTG	R: TCCATGTCAAACGTGAGCGA
SRC	F: GGTCTATGTCGAGAGCTGGC	F: AGTCCCCTGGCTCGGTTAG
	R: ATGGGGGCAAGGAAGTGATG	R: TGTCATGGCTACACAGGTCG
ABCG2	F: AACCCAGCTAGGTCAGACGA	F: GCCCTTCATTTCACGAGTGG
	R: TCGCGGTGCTCCATTTATCA	R: CCTGTGGGTCCCAGAATAGC
ABCB1	F: GCTACATGAGAGCGGAGGAC	F: TTGAAGCCGTAAGAGGCTGAG
	R: TGGATGATGGCAGCCAAAGT	R: CCCAAATACGCCAACAGCAG
MAPK1	F: TCTGTAGGCTGCATTCTGGC	F: TAAATTGGTCAGGACAAGGGCT
	R: CCCTTGCTAGAGCTCACTGT	R: GACCAGGGTAAAGAACTGGGG
MAPK8	F: CAGATTCCCCTGCTGTGGTT	F: CTTCAGAAGCAGAAGCCCCA
	R: GCTTGCTTTTGTCAGGCACA	R: TGCTGCACCTAAAGGAGACG
MAPK14	F: AGAGTCTCTGTCGACTTGCTG	F: CACAGGGACCTAAAGCCCAG
	R: TGGTGGCACAAAGCTGATGA	R: TTCTTCAGAAGCTCAGCCCC
GAPDH	F: AATGGGCAGCCGTTAGGAAA	F: CTTCTCCTGCAGCCTCGT
	R: GCGCCCAATACGACCAAATC	R: ACTGTGCCGTTGAATTTGCC

**Table 2:** Analysis of topological parameters of hub targets.

**Gene**	**UniProt ID**	**Betweenness centrality**	**Closeness centrality**	**Degree centrality**
CASP3	P42574	27.24545455	0.066666667	13
STAT3	P40763	14.08585859	0.0625	12
SRC	P12931	14.08585859	0.0625	12
ABCG2	Q9UNQ0	16.82020202	0.055555556	10
ABCB1	P08183	12.48686869	0.052631579	9

**Table 3:** Proteins and grid box centers for molecular docking.

**Name**	**Docking center (** **x**, **y**, **z)**	**Docking pocket (** **x**, **y**, **z)**
CASP3—Rivaroxaban	*x* = −3.832, *y* = −19.371, *z* = −7.103	*x* = 55.9722222222, *y* = 63.1944444444, *z* = 52.3611111111
STAT3—Rivaroxaban	*x* = −2.556, *y* = 14.534, *z* = 26.433	*x* = 80.0, *y* = 122.0, *z* = 90.0
SRC—Rivaroxaban	*x* = −6.007, *y* = −27.561, *z* = 14.642	*x* = 48.9666666667, *y* = 47.0833333333, *z* = 64.0333333333
ABCG2—Rivaroxaban	*x* = 96.631, *y* = 93.689, *z* = 102.553	*x* = 76.7666666667, *y* = 60.3166666667, *z* = 124.288888889
ABCB1—Rivaroxaban	*x* = 151.237, *y* = 149.332, *z* = 146.707	*x* = 74.0, *y* = 96.0, *z* = 102.0

**Table 4:** The binding free energy analysis of RIV and the hub targets.

**Contribution components**	**ABCB1-RIV**	**ABCG2-RIV**	**CASP3-RIV**	**SRC-RIV**	**STAT3-RIV**
Δ_VDWAALS_	−38.90 ± 0.53	−35.42 ± 0.31	−34.27 ± 0.49	−44.64 ± 0.37	−34.16 ± 0.84
ΔE_elec_	−9.87 ± 0.18	−20.13 ± 1.29	−13.25 ± 0.33	−16.17 ± 0.52	−10.54 ± 1.76
ΔE_GB_	24.49 ± 0.44	36.12 ± 0.86	29.10 ± 1.79	37.36 ± 0.79	23.60 ± 0.03
ΔE_surf_	−5.28 ± 0.10	−4.84 ± 0.00	−3.95 ± 0.07	−6.96 ± 0.03	−3.89 ± 0.07
ΔG_gas_	−48.78 ± 0.56	−55.55 ± 1.33	−47.52 ± 0.59	−60.81 ± 0.64	−44.70 ± 1.95
ΔG_solvation_	19.20 ± 0.45	31.27 ± 0.86	25.14 ± 1.79	30.40 ± 0.79	19.71 ± 0.07
ΔTotal	−29.57 ± 0.72	−24.28 ± 1.58	−22.38 ± 1.89	−30.42 ± 1.02	−24.99 ± 1.95

*Note: *Δ*G*_gas_ = Δ_VDWAALS_ + Δ*E*_elec_. Δ*G*_solvation_ = Δ*E*_surf_ + Δ*E*_surf_. ΔTotal = Δ*G*_gas_ + Δ*G*_solvation_.

Abbreviations: *E*_elec_, electrostatic energy; *E*_GB_, polar solvation energy; *E*_surf_, nonpolar solvation energy; *G*_gas_, gas-phase energy; *G*_solvation_, solvation energy; VDWALLS, Van der Waals force.

## Data Availability

Data used to support the findings of this study are available from the corresponding authors upon request.

## Supporting Information

Supporting Information 1 The original western blots were shown in the supporting information.

Supporting Information 2 The enrichment results of GO function annotation and KEGG pathway analysis are shown in the supporting tables.

## References

[1] Chu T. F., Rupnick M. A., Kerkela R. (2007). Cardiotoxicity Associated With Tyrosine Kinase Inhibitor Sunitinib. *Lancet*.

[2] Aldemir M. N., Simsek M., Kara A. V. (2020). The Effect of Adenosine Triphosphate on Sunitinib-Induced Cardiac Injury in Rats. *Human & Experimental Toxicology*.

[3] Samama M. M. (2011). The Mechanism of Action of Rivaroxaban – An Oral, Direct Factor Xa Inhibitor – Compared with Other Anticoagulants. *Thrombosis Research*.

[4] Cohen O., Levy-Mendelovich S., Ageno W. (2020). Rivaroxaban for the Treatment of Venous Thromboembolism in Pediatric Patients. *Expert Review of Cardiovascular Therapy*.

[5] Yassin A. S., Abubakar H., Mishra T. (2019). Rivaroxaban for Left Ventricular Thrombus. *American Journal of Therapeutics*.

[6] EINSTEIN–PE Investigators (2012). Oral Rivaroxaban for the Treatment of Symptomatic Pulmonary Embolism. *New England Journal of Medicine*.

[7] Zannad F., Anker S. D., Byra W. M. (2018). Rivaroxaban in Patients With Heart Failure, Sinus Rhythm, and Coronary Disease. *New England Journal of Medicine*.

[8] Guillou S., Beaumont J., Tamareille S. (2020). Direct Rivaroxaban-Induced Factor XA Inhibition Proves to Be Cardioprotective in Rats. *Shock*.

[9] Al-Harbi N. O., Imam F., Alharbi M. M. (2020). Role of Rivaroxaban in Sunitinib-Induced Renal Injuries Via Inhibition of Oxidative Stress-Induced Apoptosis and Inflammation Through the Tissue Nacrosis Factor-*α* Induced Nuclear Factor-*κ*appa B Signaling Pathway in Rats. *Journal of Thrombosis and Thrombolysis*.

[10] Qi J. Y., Yang Y. K., Jiang C. (2022). Exploring the Mechanism of Danshensu in the Treatment of Doxorubicin-Induced Cardiotoxicity Based on Network Pharmacology and Experimental Evaluation. *Frontiers in Cardiovascular Medicine*.

[11] Zhang R., Zhu X., Bai H., Ning K. (2019). Network Pharmacology Databases for Traditional Chinese Medicine: Review and Assessment. *Frontiers in Pharmacology*.

[12] Gan J., Tang F. M. K., Su X. (2019). MicroRNA-1 Inhibits Cardiomyocyte Proliferation in Mouse Neonatal Hearts by Repressing CCND1 Expression. *Annals of Translational Medicine*.

[13] van der Spoel D., Lindahl E., Hess B., Groenhof G., Mark A. E., Berendsen H. J. C. (2005). GROMACS: Fast, Flexible, and Free. *Journal of Computational Chemistry*.

[14] Wang D., Lv L., Du J., Tian K., Chen Y., Chen M. (2024). TRIM16 and PRC1 Are Involved in Pancreatic Cancer Progression and Targeted by Delphinidin. *Chemical Biology & Drug Design*.

[15] Tao J., Ni C., Jin Y. (2013). The Coexistence of Clear Cell Renal Cell Carcinoma and Gastrointestinal Stromal Tumor With Portal Vein Metastasis, and its Favorable Response to Sunitinib. *Expert Review of Anticancer Therapy*.

[16] Goodman V. L., Rock E. P., Dagher R. (2007). Approval Summary: Sunitinib for the Treatment of Imatinib Refractory or Intolerant Gastrointestinal Stromal Tumors and Advanced Renal Cell Carcinoma. *Clinical Cancer Research*.

[17] Harvey P. A., Leinwand L. A. (2015). Oestrogen Enhances Cardiotoxicity Induced by Sunitinib by Regulation of Drug Transport and Metabolism. *Cardiovascular Research*.

[18] Yang Y., Li N., Chen T. (2019). Sirt3 Promotes Sensitivity to Sunitinib-Induced Cardiotoxicity Via Inhibition of GTSP1/JNK/Autophagy Pathway In Vivo and In Vitro. *Archives of Toxicology*.

[19] Kerkela R., Woulfe K. C., Durand J. B. (2009). Sunitinib-Induced Cardiotoxicity Is Mediated by off-Target Inhibition of AMP-Activated Protein Kinase. *Clinical and Translational Science*.

[20] Yang Y., Li N., Chen T. (2019). Trimetazidine Ameliorates Sunitinib-Induced Cardiotoxicity in Mice via the AMPK/mTOR/Autophagy Pathway. *Pharmaceutical Biology*.

[21] Ren C., Sun K., Zhang Y. (2021). Sodium-Glucose CoTransporter-2 Inhibitor Empagliflozin Ameliorates Sunitinib-Induced Cardiac Dysfunction via Regulation of AMPK-mTOR Signaling Pathway-Mediated Autophagy. *Frontiers in Pharmacology*.

[22] Eldering E., Mackus W. . J. . M., Derks I. . A. . M. (2004). Apoptosis Via the B Cell Antigen Receptor Requires Bax Translocation and Involves Mitochondrial Depolarization, Cytochrome C Release, and Caspase-9 Activation. *European Journal of Immunology*.

[23] Mueck W., Stampfuss J., Kubitza D., Becka M. (2014). Clinical Pharmacokinetic and Pharmacodynamic Profile of Rivaroxaban. *Clinical Pharmacokinetics*.

[24] Eerenberg E. S., Kamphuisen P. W., Sijpkens M. K., Meijers J. C., Buller H. R., Levi M. (2011). Reversal of Rivaroxaban and Dabigatran by Prothrombin Complex Concentrate: A Randomized, Placebo-Controlled, Crossover Study in Healthy Subjects. *Circulation*.

[25] Liu J., Nishida M., Inui H. (2019). Rivaroxaban Suppresses the Progression of Ischemic Cardiomyopathy in a Murine Model of Diet-Induced Myocardial Infarction. *Journal of Atherosclerosis and Thrombosis*.

[26] Nakanishi N., Kaikita K., Ishii M. (2020). Cardioprotective Effects of Rivaroxaban on Cardiac Remodeling After Experimental Myocardial Infarction in Mice. *Circulation Reports*.

[27] Imam F., Al-Harbi N. O., Khan M. R. (2020). Protective Effect of RIVA Against Sunitinib-Induced Cardiotoxicity by Inhibiting Oxidative Stress-Mediated Inflammation: Probable Role of TGF-*β* and Smad Signaling. *Cardiovascular Toxicology*.

[28] Zhang Z., Zhang H., Li D., Zhou X., Qin Q., Zhang Q. (2021). Caspase-3-Mediated GSDME Induced Pyroptosis in Breast Cancer Cells Through the ROS/JNK Signalling Pathway. *Journal of Cellular and Molecular Medicine*.

[29] Al-Chlaihawi M., Janabi A. (2023). Azilsartan Improves Doxorubicin-Induced Cardiotoxicity Via Inhibiting Oxidative Stress, Proinflammatory Pathway, and Apoptosis. *Journal of Medicine and Life*.

[30] Wang C. Z., Guo H. Z., Leng J. Z. (2024). Exercise Preconditioning Inhibits Doxorubicin-Induced Cardiotoxicity via YAP/STAT3 Signaling. *Heliyon*.

[31] Chang W. T., Shih J. Y., Lin Y. W. (2022). Dapagliflozin Protects Against Doxorubicin-Induced Cardiotoxicity by Restoring STAT3. *Archives of Toxicology*.

[32] Harada M., Qin Y., Takano H. (2005). G-CSF Prevents Cardiac Remodeling After Myocardial Infarction by Activating the Jak-Stat Pathway in Cardiomyocytes. *Nature Medicine*.

[33] Chen H., Ma N., Xia J., Liu J., Xu Z. (2012). *β*2-Adrenergic Receptor-Induced Transactivation of Epidermal Growth Factor Receptor and Platelet-Derived Growth Factor Receptor via Src Kinase Promotes Rat Cardiomyocyte Survival. *Cell Biology International*.

[34] Elmadani M., Raatikainen S., Mattila O. (2023). Dasatinib Targets c-Src Kinase in Cardiotoxicity. *Toxicology Reports*.

[35] Kobashigawa L. C., Xu Y. C., Padbury J. F., Tseng Y. T., Yano N. (2014). Metformin Protects Cardiomyocyte From Doxorubicin Induced Cytotoxicity Through an AMP-Activated Protein Kinase Dependent Signaling Pathway: An In Vitro Study. *PLoS One*.

[36] Meissner K., Heydrich B., Jedlitschky G. (2006). The ATP-Binding Cassette Transporter ABCG2 (BCRP), a Marker for Side Population Stem Cells, Is Expressed in Human Heart. *Journal of Histochemistry & Cytochemistry*.

[37] Schumacher T., Benndorf R. A. (2017). ABC Transport Proteins in Cardiovascular Disease-A Brief Summary. *Molecules*.

[38] Watanabe A., Yamamoto K., Ioroi T. (2017). Association of Single Nucleotide Polymorphisms in STAT3, ABCB1, and ABCG2 With Stomatitis in Patients With Metastatic Renal Cell Carcinoma Treated With Sunitinib: A Retrospective Analysis in Japanese Patients. *Biological and Pharmaceutical Bulletin*.

[39] Sun F., Chen Z., Yao P., Weng B., Liu Z., Cheng L. (2021). Meta-Analysis of ABCG2 and ABCB1 Polymorphisms With Sunitinib-Induced Toxicity and Efficacy in Renal Cell Carcinoma. *Frontiers in Pharmacology*.

[40] Higashikuni Y., Sainz J., Nakamura K. (2012). The ATP-Binding Cassette Transporter ABCG2 Protects Against Pressure Overload-Induced Cardiac Hypertrophy and Heart Failure by Promoting Angiogenesis and Antioxidant Response. *Arteriosclerosis, Thrombosis, and Vascular Biology*.

[41] Durmus S., Naik J., Buil L., Wagenaar E., van Tellingen O., Schinkel A. H. (2014). *In Vivo* Disposition of Doxorubicin Is Affected by Mouse Oatp1a/1b and Human OATP1A/1B Transporters. *International Journal of Cancer*.

[42] Cargnello M., Roux P. P. (2011). Activation and Function of the MAPKs and their Substrates, the MAPK-Activated Protein Kinases. *Microbiology and Molecular Biology Reviews*.

[43] Yang N., Zou C., Luo W. (2023). Sclareol Attenuates Angiotensin II-Induced Cardiac Remodeling and Inflammation Via Inhibiting MAPK Signaling. *Phytotherapy Research*.

[44] Kojonazarov B., Novoyatleva T., Boehm M. (2017). p38 MAPK Inhibition Improves Heart Function in Pressure-Loaded Right Ventricular Hypertrophy. *American Journal of Respiratory Cell and Molecular Biology*.

[45] Sarawi W. S., Alhusaini A. M., Fadda L. M. (2021). Nano-Curcumin Prevents Cardiac Injury, Oxidative Stress and Inflammation, and Modulates TLR4/NF-*κ*B and MAPK Signaling in Copper Sulfate-Intoxicated Rats. *Antioxidants*.

[46] Zheng H., Xu N., Zhang Z., Wang F., Xiao J., Ji X. (2022). Setanaxib (GKT137831) Ameliorates Doxorubicin-Induced Cardiotoxicity by Inhibiting the NOX1/NOX4/Reactive Oxygen Species/MAPK Pathway. *Frontiers in Pharmacology*.

[47] Chen L., Sun X., Wang Z. (2024). Resveratrol Protects Against Doxorubicin-Induced Cardiotoxicity by Attenuating Ferroptosis Through Modulating the MAPK Signaling Pathway. *Toxicology and Applied Pharmacology*.

[48] Korashy H. M., Al-Suwayeh H. A., Maayah Z. H., Ansari M. A., Ahmad S. F., Bakheet S. A. (2015). Mitogen-Activated Protein Kinases Pathways Mediate the Sunitinib-Induced Hypertrophy in Rat Cardiomyocyte H9c2 Cells. *Cardiovascular Toxicology*.

[49] Yue J., López J. M. (2020). Understanding MAPK Signaling Pathways in Apoptosis. *International Journal of Molecular Sciences*.

[50] Alvarado-Kristensson M., Melander F., Leandersson K., Rönnstrand L., Wernstedt C., Andersson T. (2004). p38-MAPK Signals Survival by Phosphorylation of Caspase-8 and Caspase-3 in Human Neutrophils. *Journal of Experimental Medicine*.

[51] Zeng J. J., Shi H. Q., Ren F. F. (2023). Notoginsenoside R1 Protects Against Myocardial Ischemia/Reperfusion Injury in Mice via Suppressing TAK1-JNK/p38 Signaling. *Acta Pharmacologica Sinica*.

[52] Xuan T., Wang D., Lv J. (2020). Downregulation of Cypher Induces Apoptosis in Cardiomyocytes via Akt/p38 MAPK Signaling Pathway. *International Journal of Medical Sciences*.

[53] Xiong W., Chen H., Lu J. (2021). IL-39 Increases ROS Production and Promotes the Phosphorylation of p38 MAPK in the Apoptotic Cardiomyocytes. *Folia Histochemica et Cytobiologica*.

[54] Hashikata T., Yamaoka-Tojo M., Namba S. (2015). Rivaroxaban Inhibits Angiotensin II-Induced Activation in Cultured Mouse Cardiac Fibroblasts Through the Modulation of NF-*κ*B Pathway. *International Heart Journal*.

[55] Ghafouri-Fard S., Khanbabapour Sasi A., Hussen B. M. (2022). Interplay Between PI3K/AKT Pathway and Heart Disorders. *Molecular Biology Reports*.

[56] Chen Y., Chen C., Fang J. (2022). Targeting the Akt/PI3K/mTOR Signaling Pathway for Complete Eradication of Keloid Disease by Sunitinib. *Apoptosis*.

[57] Fan X., Tong Y., Chen Y., Chen Y. (2022). Sunitinib Reduced the Migration of Ectopic Endometrial Cells via P-VEGFR-PI3K-AKT-YBX1-Snail Signaling Pathway. *Analytical Cellular Pathology*.

[58] Aksamitiene E., Kiyatkin A., Kholodenko B. N. (2012). Cross-Talk Between Mitogenic Ras/MAPK and Survival PI3K/Akt Pathways: A Fine Balance. *Biochemical Society Transactions*.

[59] Zhou J., Du T., Li B., Rong Y., Verkhratsky A., Peng L. (2015). Crosstalk Between MAPK/ERK and PI3K/AKT Signal Pathways During Brain Ischemia/Reperfusion. *ASN Neuro*.

[60] Sun S., Wang L., Wang J. (2023). Maresin1 Prevents Sepsis-Induced Acute Liver Injury by Suppressing NF-*κ*B/Stat3/MAPK Pathways, Mitigating Inflammation. *Heliyon*.

[61] Zhou L., Ni C., Liao R. (2024). Activating SRC/MAPK Signaling via 5-HT1A Receptor Contributes to the Effect of Vilazodone on Improving Thrombocytopenia. *Elife*.

[62] Zhong T., Feng M., Su M. (2021). Qihuzha Granule Attenuated LPS-Induced Acute Spleen Injury in Mice via Src/MAPK/Stat3 Signal Pathway. *Journal of Ethnopharmacology*.

