# Supplementary material for: Rivaroxaban Ameliorates Sunitinib-Induced Injury of Cardiomyocytes via Repressing MAPK Signaling Pathway
Source: Cardiovasc Ther. 2025 Jul 25;2025:2208110. doi: 10.1155/cdr/2208110 (PMC12316504; doi:10.1155/cdr/2208110)
Supplement: Supporting Information 1 — The original western blots were shown in the supporting information. [file 2208110.f1.docx]

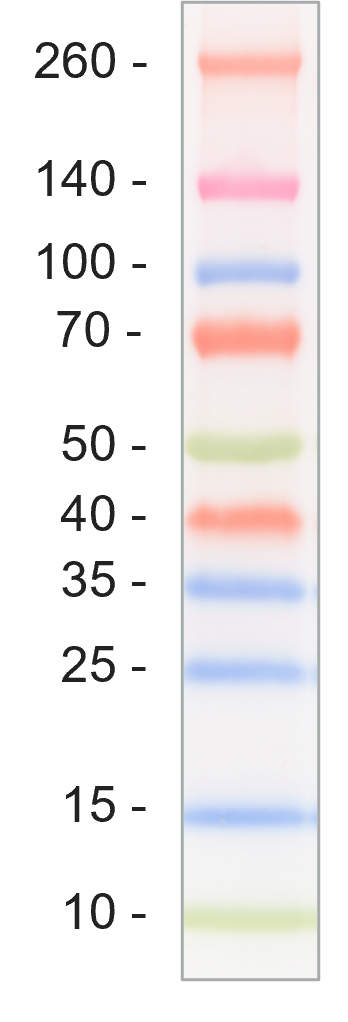


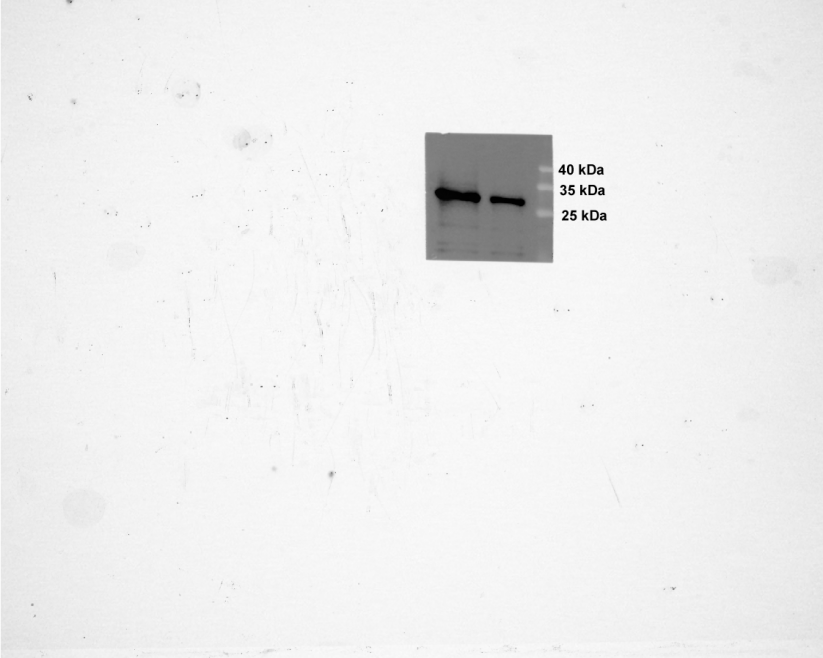


Original image of Figure 1F Bcl-2


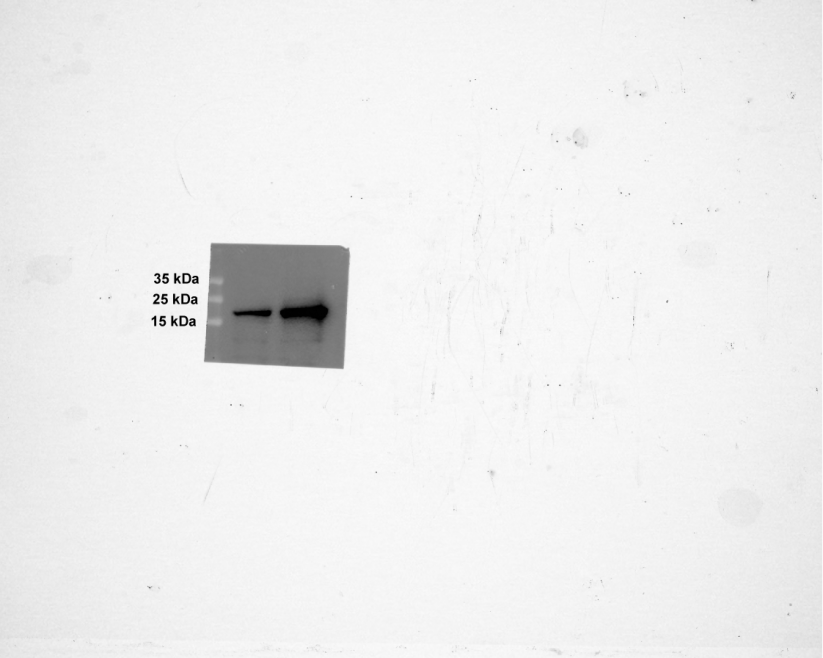


Original image of Figure 1F Bax


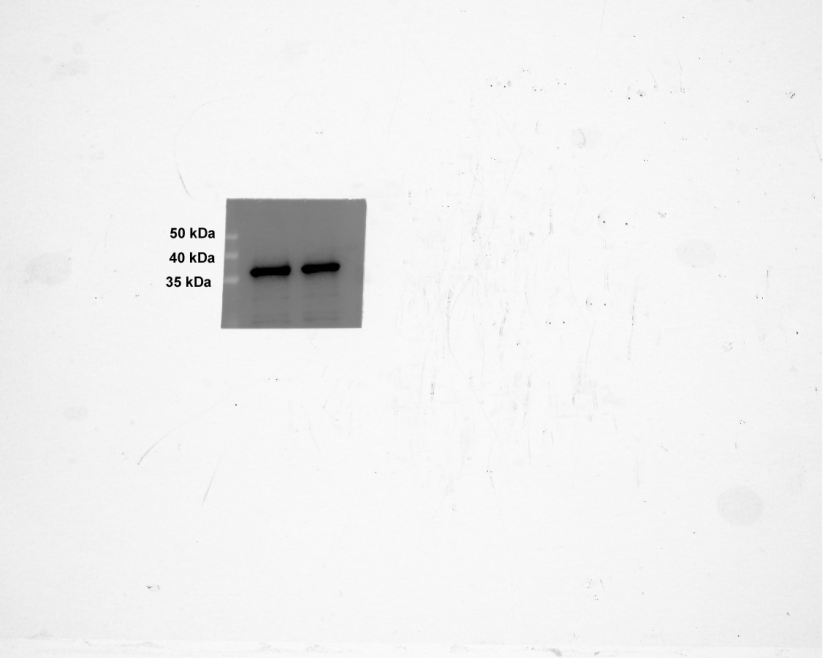


Original image of Figure 1F GAPDH


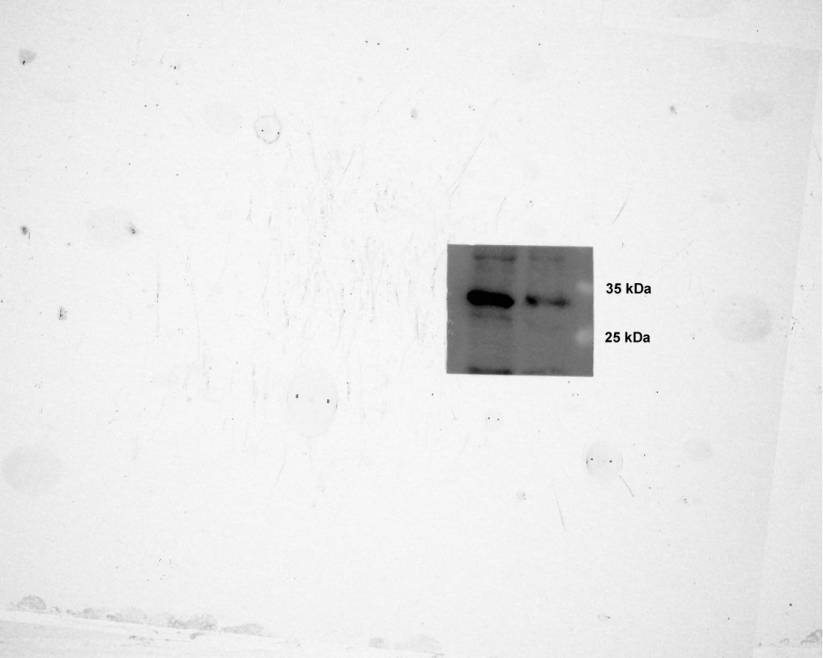


Original image of Figure 1G Bcl-2


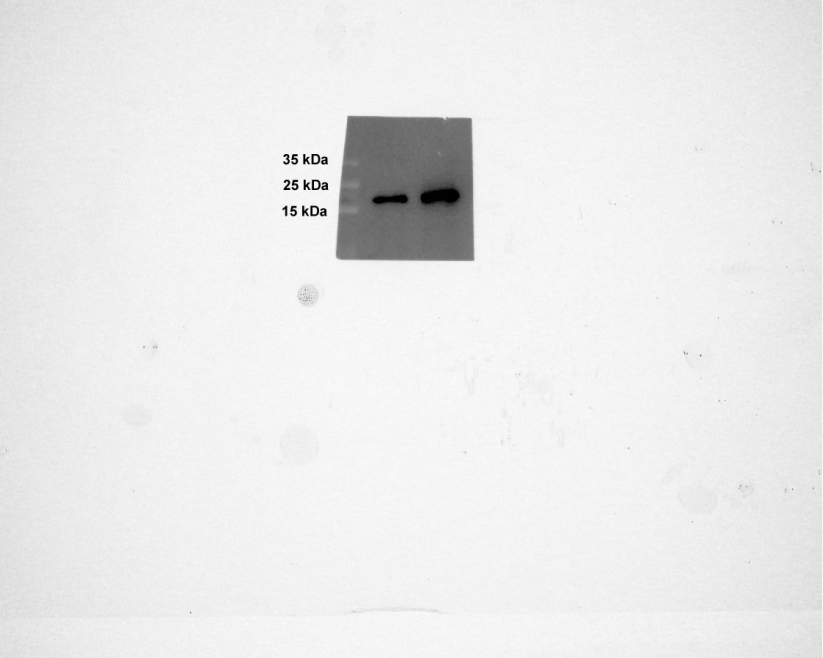


Original image of Figure 1G Bax


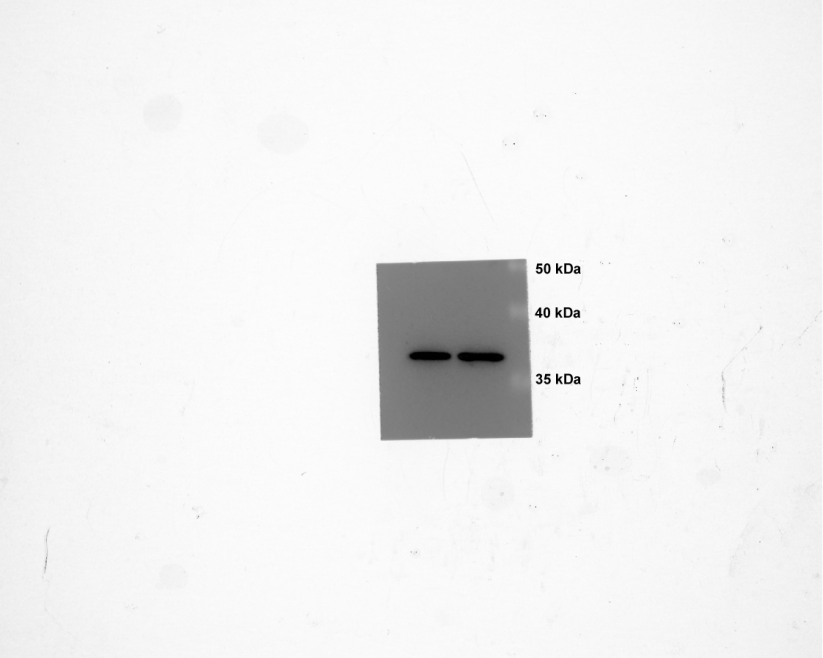


Original image of Figure 1G GAPDH


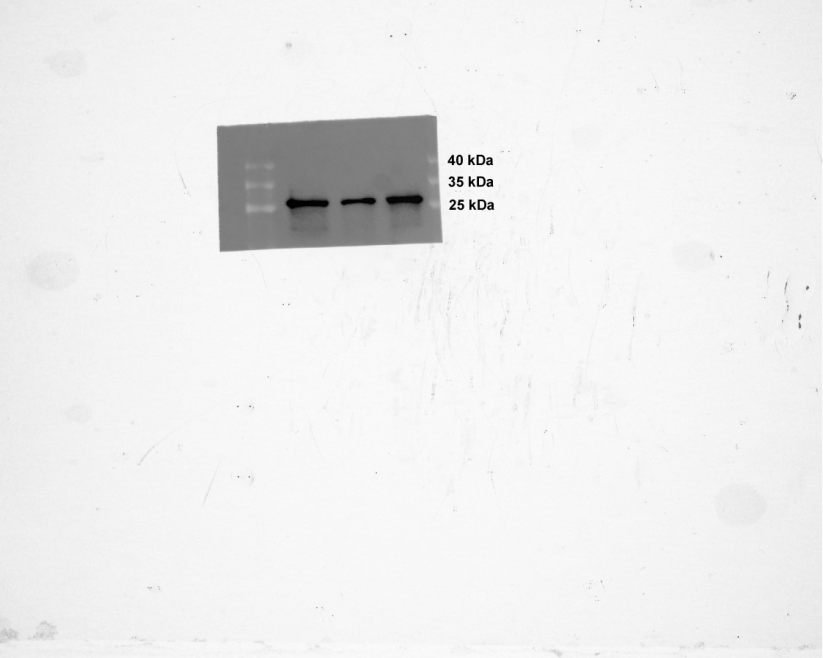


Original image of Figure 2H Bcl-2


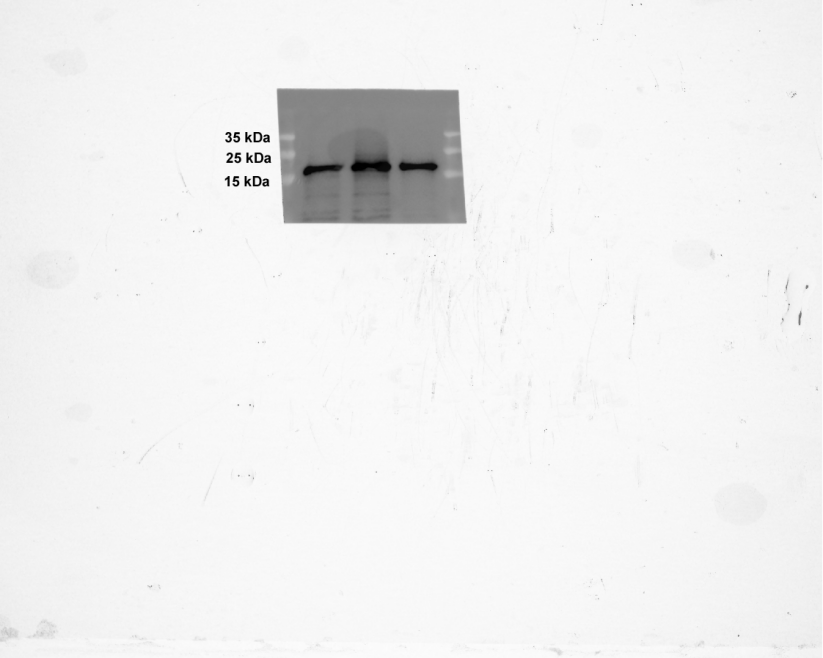


Original image of Figure 2H Bax


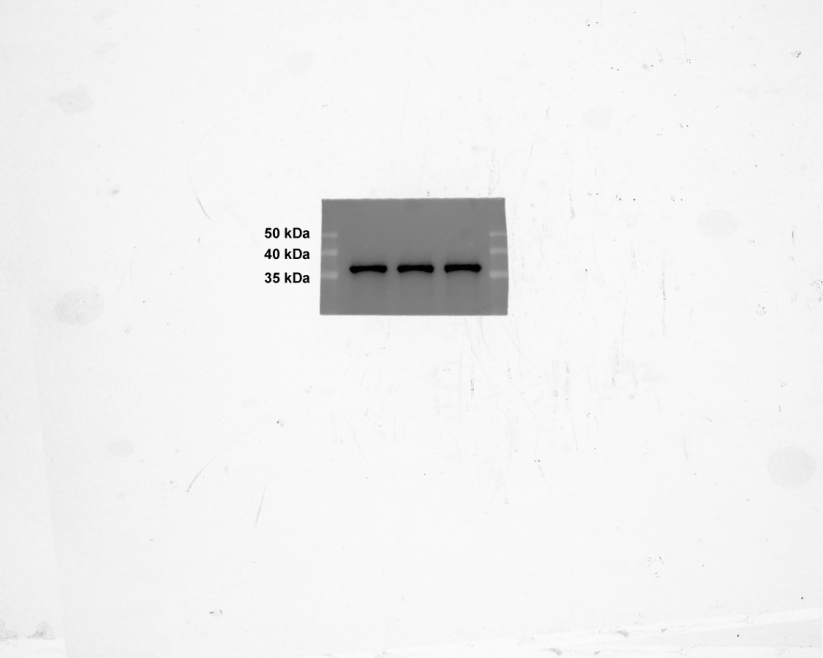


Original image of Figure 2H GAPDH


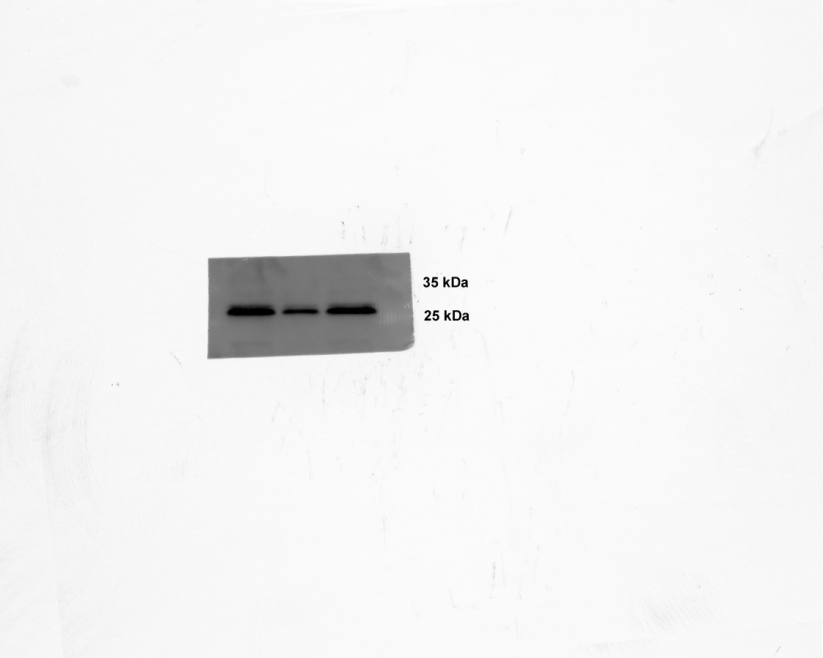


Original image of Figure 2I Bcl-2


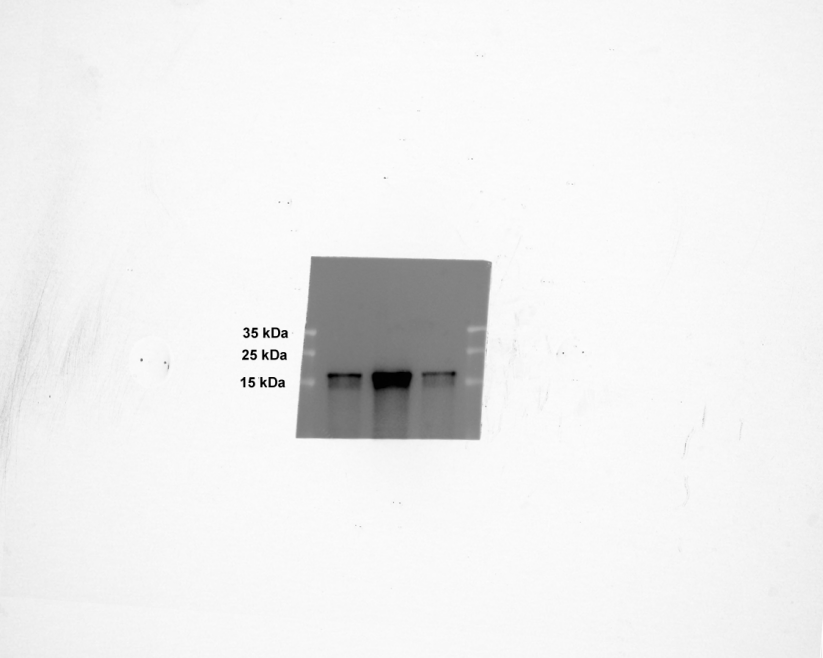


Original image of Figure 2I Bax


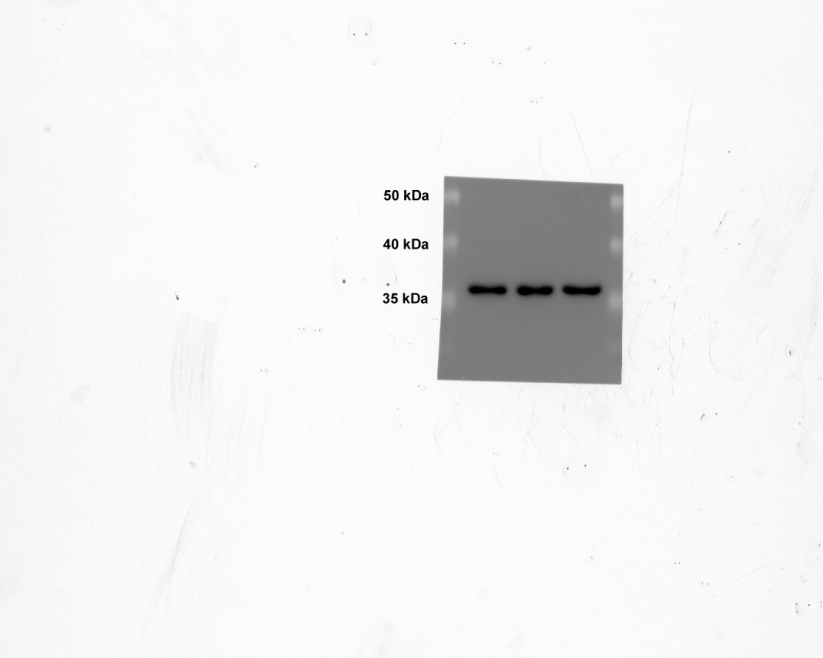


Original image of Figure 2I GAPDH


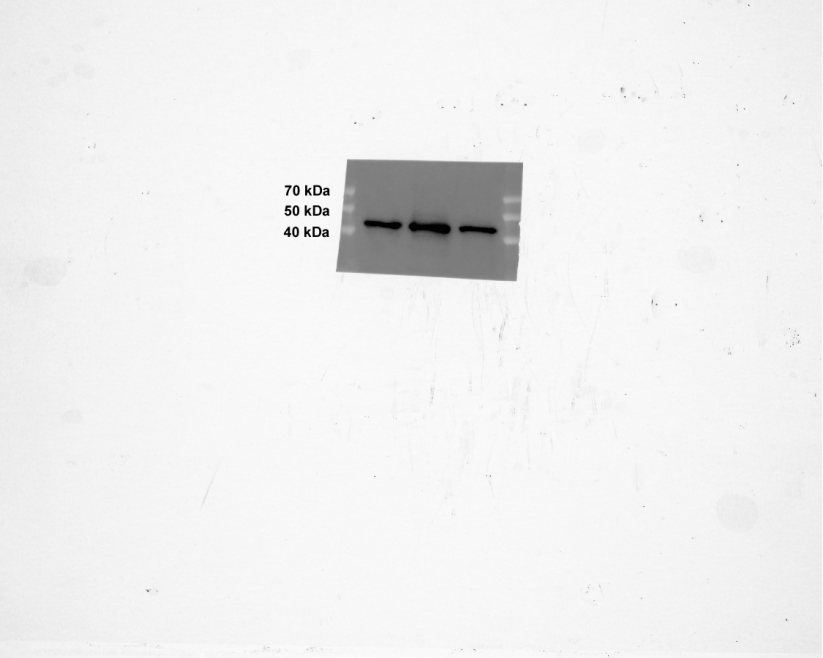


Original image of Figure 6I p-ERK1/2


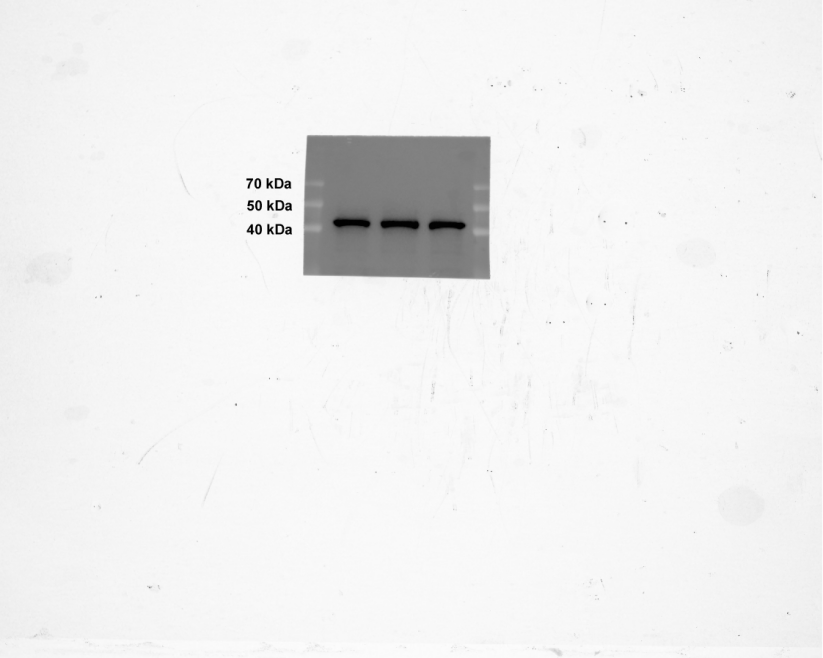


Original image of Figure 6I ERK1/2


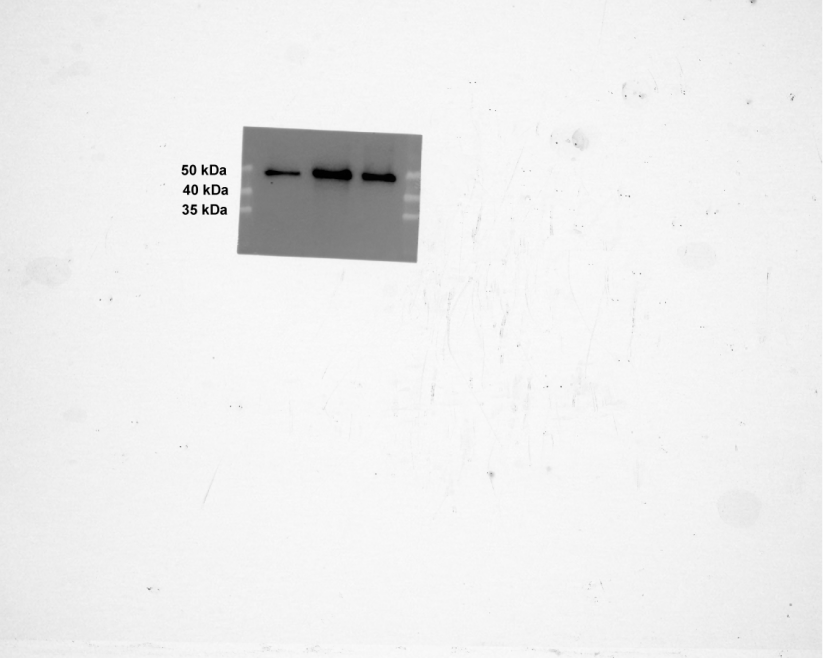


Original image of Figure 6I p-JNK


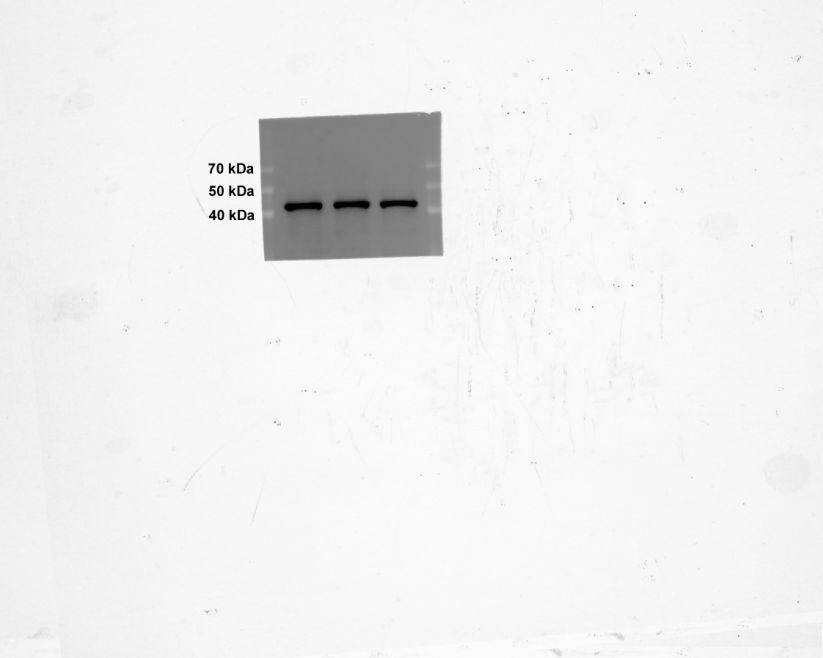


Original image of Figure 6I JNK


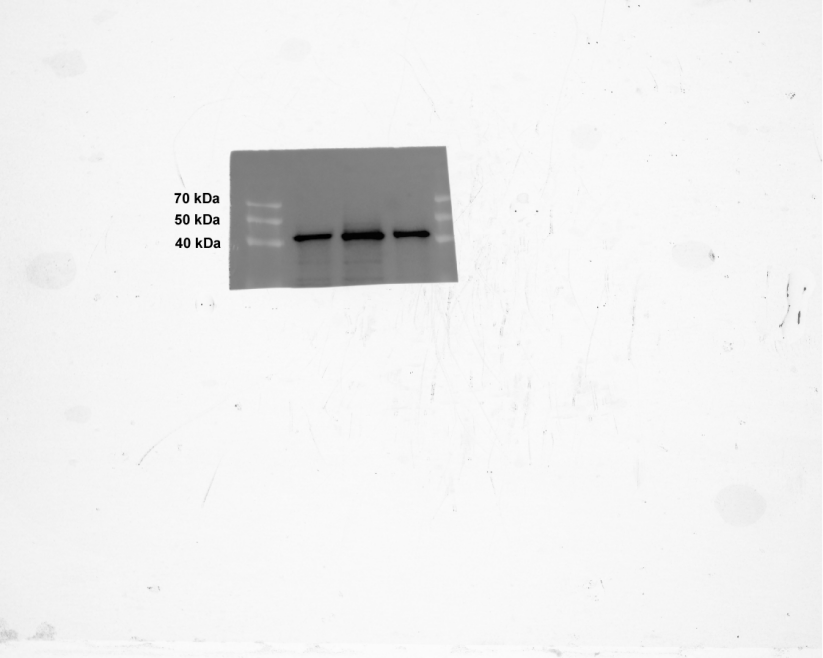


Original image of Figure 6I p-p38


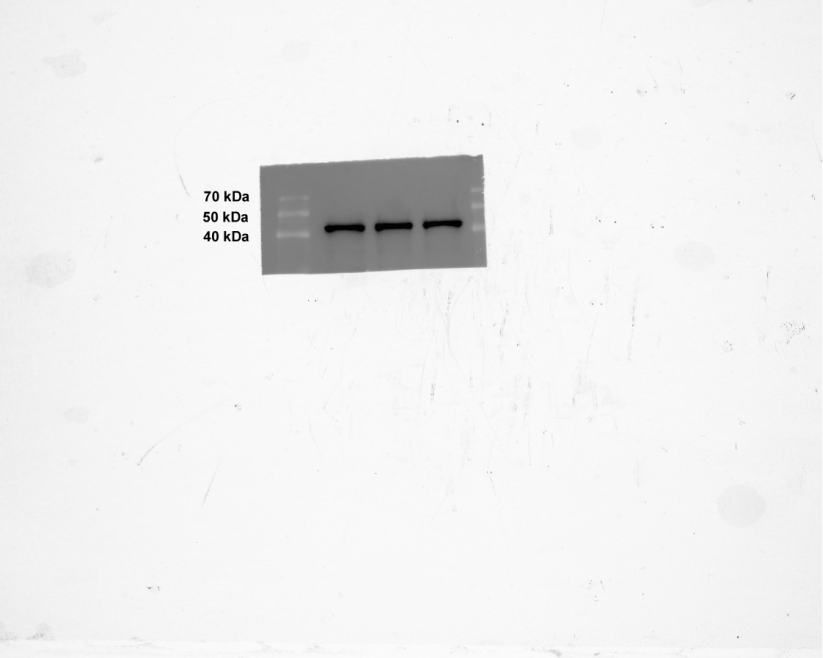


Original image of Figure 6I p38


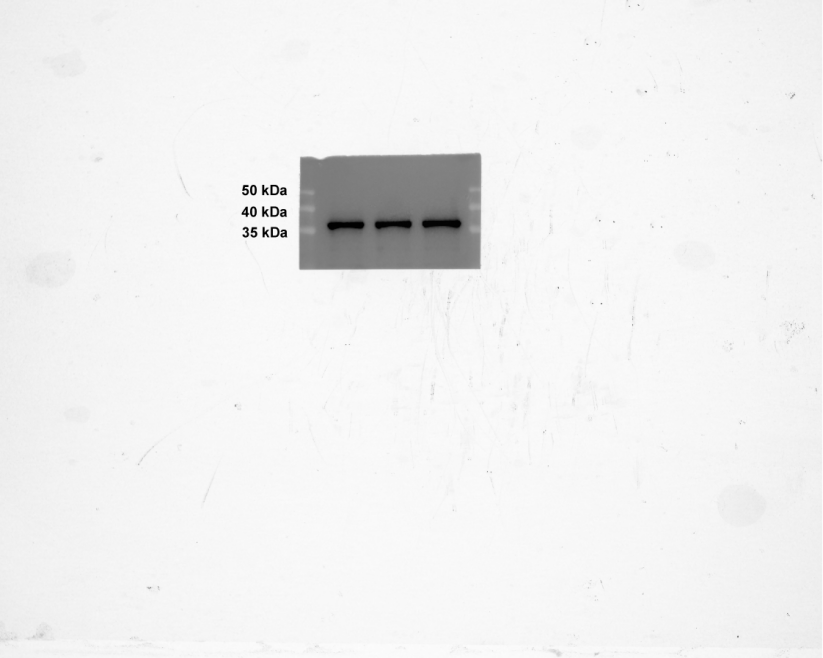


Original image of Figure 6I GAPDH


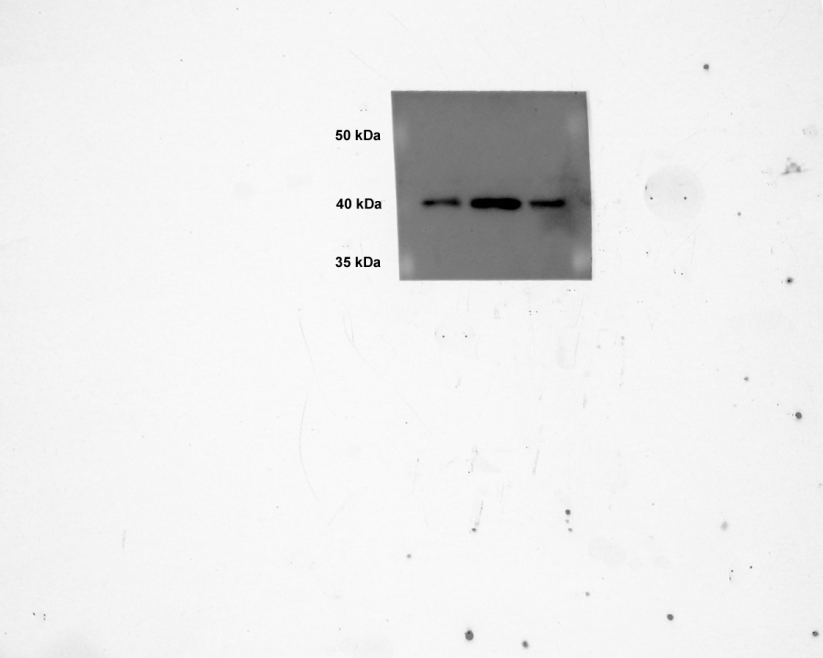


Original image of Figure 6J p-ERK1/2


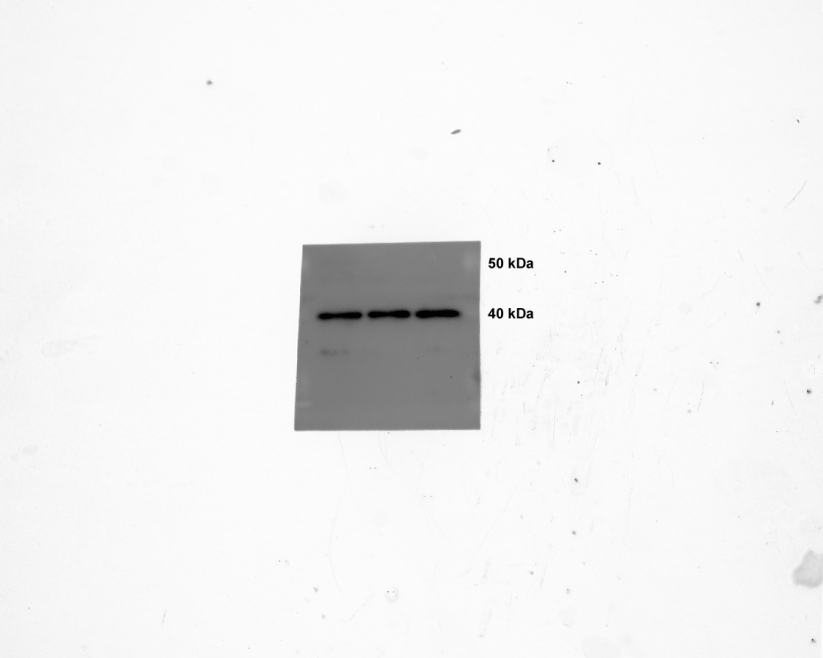


Original image of Figure 6J ERK1/2


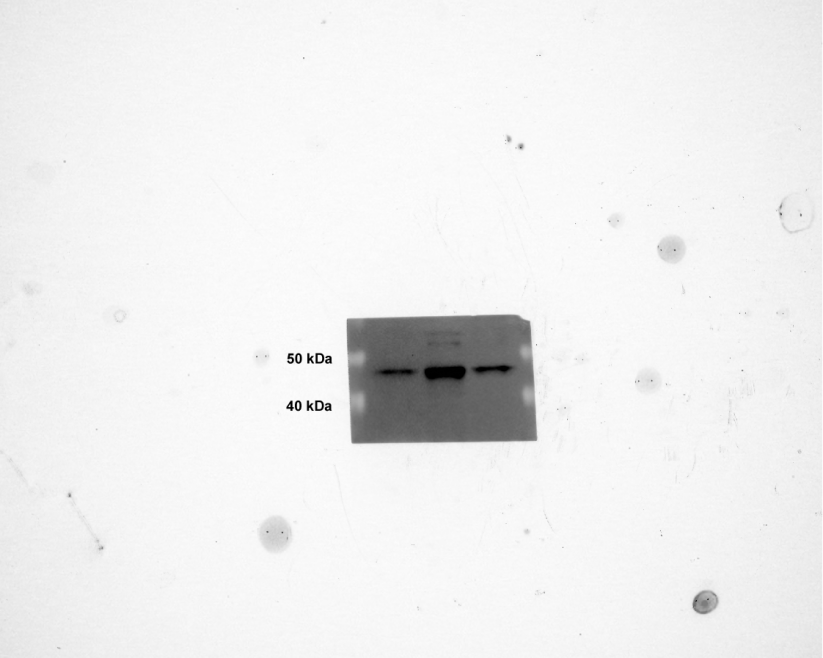


Original image of Figure 6J p-JNK


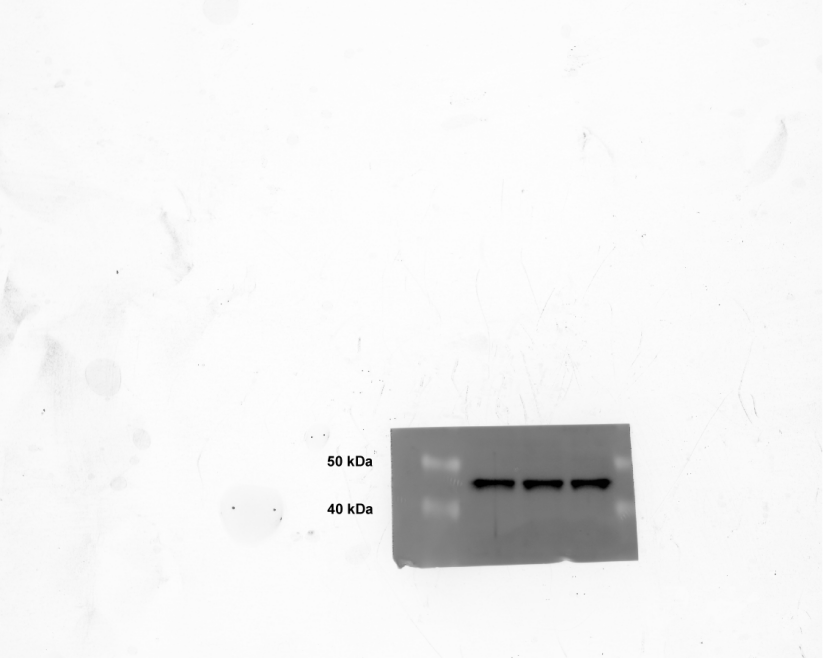


Original image of Figure 6J JNK


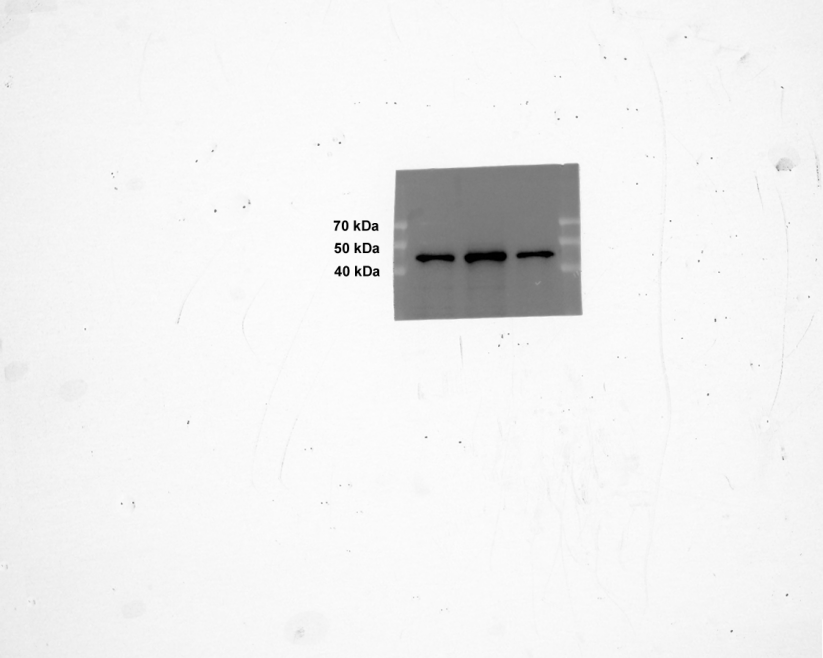


Original image of Figure 6J p-p38


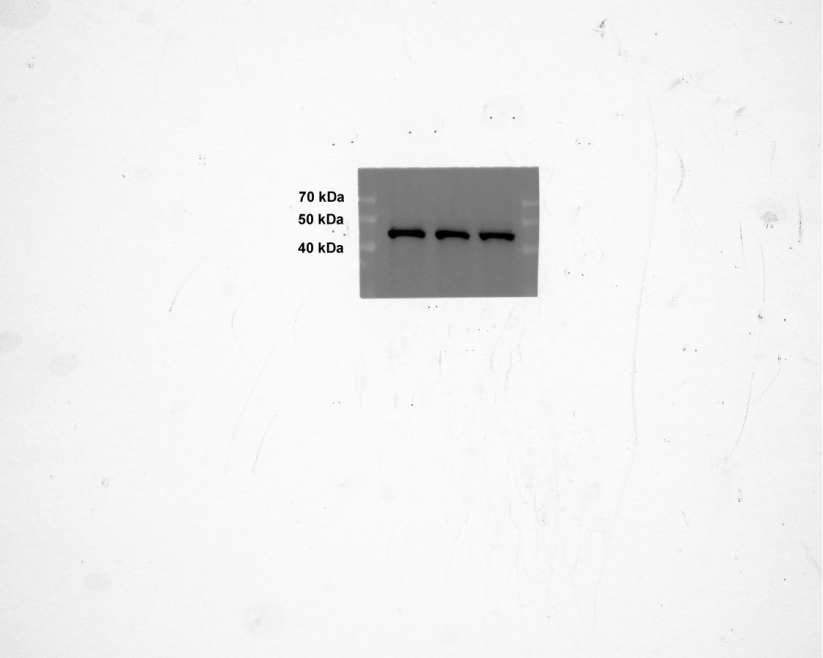


Original image of Figure 6J p38


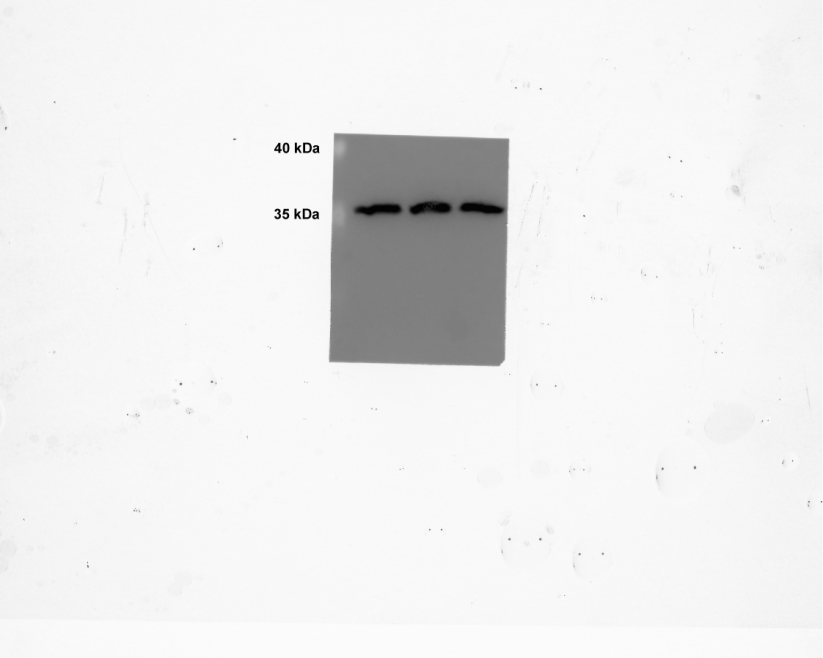


Original image of Figure 6J GAPDH
